# MicroRNA-181b Controls Atherosclerosis and Aneurysms Through Regulation of TIMP-3 and Elastin

**DOI:** 10.1161/CIRCRESAHA.116.309321

**Published:** 2017-01-05

**Authors:** Karina Di Gregoli, Nur Najmi Mohamad Anuar, Rosaria Bianco, Stephen J. White, Andrew C. Newby, Sarah J. George, Jason L. Johnson

**Affiliations:** From the Laboratory of Cardiovascular Pathology, School of Clinical Sciences, University of Bristol, England.

**Keywords:** aortic aneurysm, abdominal, atherosclerosis, macrophages, microRNAs, tissue inhibitor of metalloproteinase-3

## Abstract

**Rationale:**

Atherosclerosis and aneurysms are leading causes of mortality worldwide. MicroRNAs (miRs) are key determinants of gene and protein expression, and atypical miR expression has been associated with many cardiovascular diseases; although their contributory role to atherosclerotic plaque and abdominal aortic aneurysm stability are poorly understood.

**Objective:**

To investigate whether miR-181b regulates tissue inhibitor of metalloproteinase-3 expression and affects atherosclerosis and aneurysms.

**Methods and Results:**

Here, we demonstrate that miR-181b was overexpressed in symptomatic human atherosclerotic plaques and abdominal aortic aneurysms and correlated with decreased expression of predicted miR-181b targets, tissue inhibitor of metalloproteinase-3, and elastin. Using the well-characterized mouse atherosclerosis models of *Apoe*^−^^/−^ and *Ldlr*^−/−^, we observed that in vivo administration of locked nucleic acid anti-miR-181b retarded both the development and the progression of atherosclerotic plaques. Systemic delivery of anti-miR-181b in angiotensin II–infused *Apoe*^−/−^ and *Ldlr*^−/−^ mice attenuated aneurysm formation and progression within the ascending, thoracic, and abdominal aorta. Moreover, miR-181b inhibition greatly increased elastin and collagen expression, promoting a fibrotic response and subsequent stabilization of existing plaques and aneurysms. We determined that miR-181b negatively regulates macrophage tissue inhibitor of metalloproteinase-3 expression and vascular smooth muscle cell elastin production, both important factors in maintaining atherosclerotic plaque and aneurysm stability. Validation studies in *Timp3*^−/−^ mice confirmed that the beneficial effects afforded by miR-181b inhibition are largely tissue inhibitor of metalloproteinase-3 dependent, while also revealing an additional protective effect through elevating elastin synthesis.

**Conclusions:**

Our findings suggest that the management of miR-181b and its target genes provides therapeutic potential for limiting the progression of atherosclerosis and aneurysms and protecting them from rupture.

## Abstract 2

Supplemental Digital Content is available in the text.

Atherosclerosis and aneurysms are the leading causes of cardiovascular disease, the major source of mortality and morbidity worldwide.^1^ Unstable atherosclerotic plaques are characterized by a relative preponderance of inflammatory cells and heightened proteolytic activity.^2,3^ Clinically relevant abdominal aortic aneurysms (AAAs) are also associated with inflammation and matrix degradation.^4^ Subsets of proinflammatory macrophages are considered central to the pathogenesis of the above inflammatory cardiovascular diseases, in part, through augmented release of matrix-degrading proteases and by decreased expression of their inhibitors. However, other macrophage subsets may play beneficial roles, for example, by facilitating smooth muscle cell recruitment, regulating neovascularization, and promoting extracellular matrix (ECM) formation/deposition.^5^ Similarly, matrix metalloproteinases (MMPs), a group of proteases produced by macrophages and abundant in pathological cardiovascular tissues,^6^ may also play a dual role. MMP knockout mice and other transgenic models show clear effects of individual MMPs on vascular repair and fibrous cap formation on the one hand or ECM destruction and hence destabilization of atherosclerotic lesions and aneurysm rupture on the other. These opposing effects of different MMPs probably underlie the disappointing results achieved with broad-spectrum MMP inhibitors in these pathologies.^7^ However, altering levels of tissue inhibitors of metalloproteinases (TIMPs) have revealed more consistent protective effects against atherosclerosis and aneurysm formation.^7^ Hence, understanding and manipulating the factors that regulate TIMP production in macrophages may provide new therapeutic avenues.

**Editorial, see p 5**

**In This Issue, see p 2**

Previous studies from our laboratory identified a distinct subpopulation of macrophages with reduced TIMP-3 expression and a concomitant increase in MMP-14 protein level and activity and associated proteolysis.^8^ TIMP-3–negative macrophages are more invasive, more susceptible to apoptosis, and more proliferative compared with the TIMP-3–positive macrophage subpopulation^8^ and are more abundant in unstable than stable plaques.^9^ Further analysis revealed that granulocyte macrophage colony-stimulating factor (GM-CSF) increased MMP-14 protein but not mRNA level by decreasing expression of microRNA (miR)-24, resulting in increased MMP-14–dependent proteolytic activity.^10^ GM-CSF consequently promoted a more invasive and proapoptotic macrophage phenotype, which was associated with unstable atherosclerotic plaques.^10^ Some evidence suggested that macrophage TIMP-3 expression may also be subjected to post-transcriptional regulation.^8,9^ MiRs are a group of noncoding RNAs able to finely regulate the protein expression of their targets through degradation of the mRNA or inhibition of protein translation. MiRs control several processes known to be involved with the initiation and progression of numerous cardiovascular diseases, such as atherosclerosis^11^ and AAAs.^12^ In particular, miR-181b is a highly conserved miR able to directly target TIMP-3 mRNA and repress TIMP-3 protein expression in hepatocarcinoma cells.^13^ In addition, miR-181b is downregulated during macrophage colony-stimulating factor (M-CSF) maturation of macrophages,^10^ whereas TIMP-3 protein and mRNA expression is enhanced.^14^ TIMP-3 has been identified in atherosclerotic plaque macrophages between the necrotic/lipid-rich core and the protective fibrous cap.^14^ Consequently, TIMP-3 may play a protective role against plaque rupture through suppressing local proteolysis. TIMP-3 mRNA levels are also increased in patients with ascending aortic aneurysms, whereas other TIMPs are not altered.^15^ Similarly, a significant association between polymorphisms of TIMP-3, but not TIMP-1 or TIMP-2, exists in patients with AAA and a positive family history of AAA.^16^ Also, aorta wall TIMP-3 expression is reduced in Marfan syndrome patients who experience an increased rate of aortic rupture.^17^

Hence, in the current study, we investigated whether miR-181b regulates TIMP-3 protein expression in atherosclerosis and AAA and whether miR-181b inhibition can ameliorate plaque and aneurysm progression. Consequently, we demonstrate for the first time that miR-181b inhibition promotes a stable plaque phenotype by restoring TIMP-3 expression in macrophages, which stabilizes AAAs by promoting collagen accumulation. Inhibition of miR-181b also directly enhances elastin deposition. This dual beneficial role of miR-181b inhibition provides substantial evidence for this approach as a therapeutic for atherosclerosis-related cardiovascular diseases, including aneurysms.

## Methods

Methods are available in the Online Data Supplement.

## Results

### MiR-181b Regulates Macrophage TIMP-3 Expression and Associates With Cardiovascular Disease Progression in Humans

**Figure 1.**
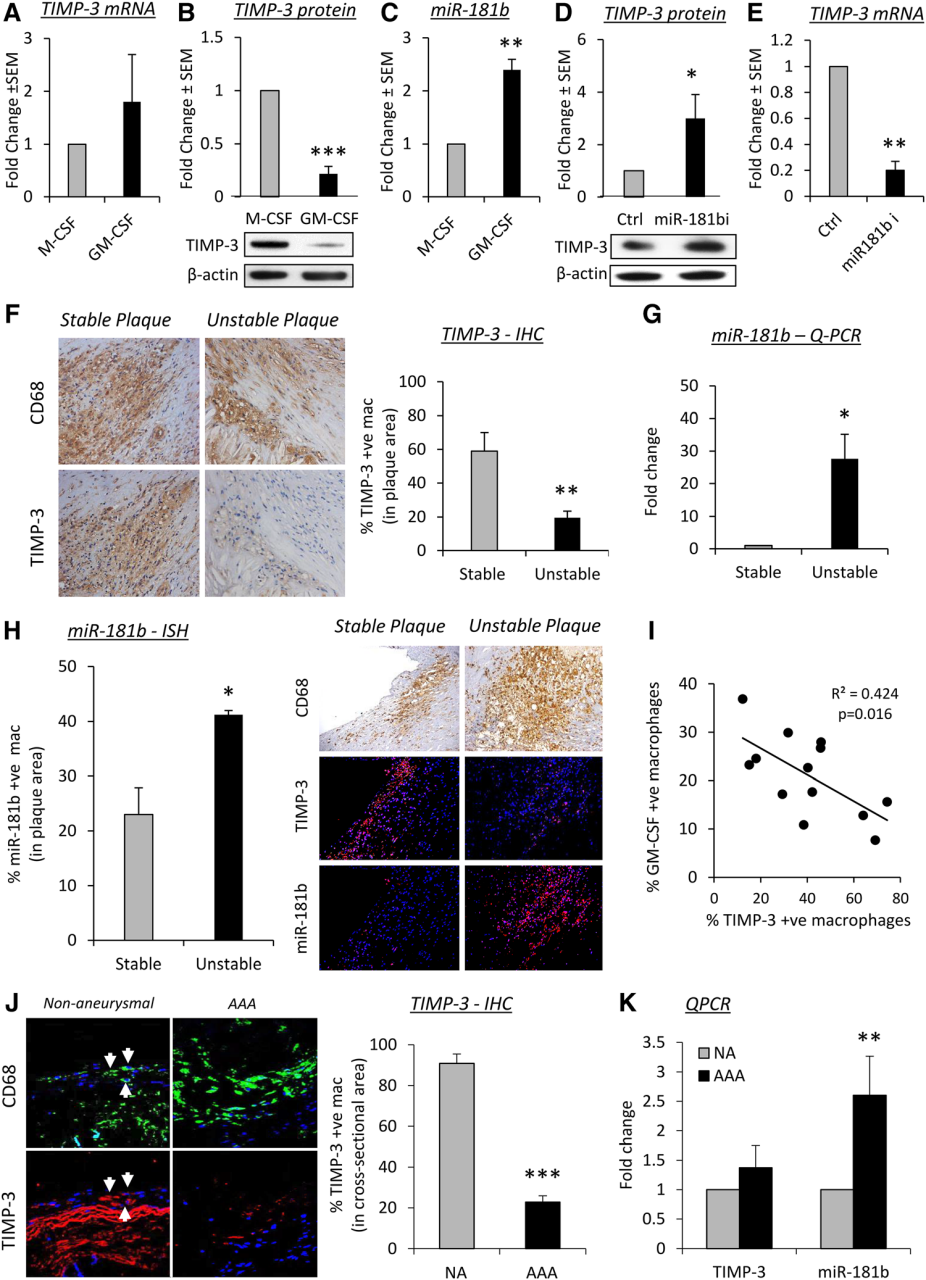
**MicroRNA (miR)-181b regulates macrophage tissue inhibitor of metalloproteinase (TIMP)-3 expression and associates with cardiovascular disease progression in humans. A**, Quantitative polymerase chain reaction (QPCR) and (**B**) Western blot of *TIMP3* mRNA and protein expression, respectively, in human macrophages differentiated in the presence of macrophage colony-stimulating factor (M-CSF) or granulocyte/macrophage colony-stimulating factor (GM-CSF), n=6/group, ***P<0.001, 2-tailed Student *t* test. **C**, QPCR of miR-181b in human macrophages differentiated in the presence of M-CSF or GM-CSF, n=6/group, **P<0.01, 2-tailed Student *t* test. **D**, Western blot and (**E**) QPCR of *TIMP3* in 7-day GM-CSF–differentiated macrophages after addition of an miR-181b inhibitor (miR-181bi) or a scrambled control (Ctrl), n=4/group, *P<0.05 and **P<0.05, 2-tailed Student *t* test. **F**, Representative images of CD68 (macrophages) and TIMP-3 protein expression by immunohistochemistry (IHC) and quantification from human stable and unstable coronary atherosclerotic plaques, n=10/group, **P<0.01, 2-tailed Student *t* test. **G**, QPCR of miR-181b expression from stable and unstable coronary atherosclerotic plaques, n=10/group, *P<0.05, 2-tailed Student *t* test. **H**, Representative images and quantification of TIMP-3 protein expression by IHC and miR-181b by in situ hybridization (ISH) from stable and unstable coronary atherosclerotic plaques, n=10/group, *P<0.05, 2-tailed Student *t* test. **I**, Correlation of TIMP-3 and GM-CSF–positive macrophages in human coronary artery atherosclerotic plaques, n=16, Spearman correlation test. **J**, Representative images of CD68 (macrophages) and TIMP-3 protein expression by IHC and quantification from control human nonaneurysmal (NA) aorta and abdominal aortic aneurysm (AAA), n=10/group, ***P<0.00315, 2-tailed Student *t* test. **K**, QPCR of TIMP-3 and miR-181b expression from control human NA aorta and AAA, n=10/group, **P<0.01, 2-tailed Student *t* test. In all cases, data represent the mean±SEM.

We first measured TIMP-3 levels in macrophages, differentiated with M-CSF or GM-CSF, respectively.^18^ Although no difference in *TIMP3* mRNA expression was detected (Figure 1A), protein levels were markedly reduced in GM-CSF–differentiated macrophages (Figure 1B). Accordingly, the level of miR-181b was significantly increased after GM-CSF macrophage differentiation compared with M-CSF macrophages (Figure 1C), implying that it could be responsible for the fall in TIMP-3 protein. To confirm this, we deployed a loss of function strategy in GM-CSF macrophages, revealing that miR-181b inhibition restored TIMP-3 protein expression to comparable levels found in M-CSF macrophages (Figure 1D), whereas the mRNA level was significantly reduced (Figure 1E), implying restored *TIMP3* translation. Hence, our findings confirm TIMP-3 as an miR-181b target^13^ and demonstrate that miR-181b serves as an important inhibitor of macrophage TIMP-3 protein expression, which is divergently regulated by colony-stimulating factors. Moreover, these changes are independent of potential regulation by MMP-14 expression/activity, which we have previously shown to be upregulated in macrophages on GM-CSF stimulation^10^ or through regulation of MMP-14 expression by miR-181b or TIMP-3 (Online Figure I).

To validate our findings in human cardiovascular pathologies, we investigated the expression of miR-181b and its putative target TIMP-3 in human coronary atherosclerotic plaques and AAAs. We observed a decreased proportion of TIMP-3–positive macrophages (CD68^+ve^ cells) in human coronary artery atherosclerotic plaques characterized as unstable compared with stable lesions (by 67%; *P*<0.01; Figure 1F), whereas no change in *TIMP3* mRNA expression was detected (Online Figure II). Furthermore, miR-181b expression in atherosclerotic plaques was inversely related to TIMP-3 protein expression because unstable plaques contained higher miR-181b levels (as assessed by quantitative polymerase chain reaction) than stable plaques (28-fold; *P*<0.05; Figure 1G). Moreover, confirmatory in situ hybridization indicated that the proportion of macrophages expressing miR-181b was significantly higher in unstable plaques compared with stable plaques (*P*<0.05; Figure 1H and Online Figure III) in direct contrast to TIMP-3 protein expression (Figure 1E). We previously showed that unstable human coronary plaques harbor a heightened proportion of GM-CSF than M-CSF–positive macrophage.^10^ We now show that the density of intraplaque TIMP-3–positive macrophages negatively correlated with the number of GM-CSF–positive macrophages in advanced plaques (*r*^2^=0.424; *P*=0.016; Figure 1I).

Similar findings were apparent in human aortic aneurysm tissues. Whereas the majority of macrophages (CD68^+ve^ cells) in nonaneurysmal aortic tissue were TIMP-3 positive (91%; Figure 1J), only 23% of macrophages within abdominal aortic tissues expressed TIMP-3 (Figure 1J). These differences were independent of alterations in mRNA expression (Figure 1K), but consistent with the significant change in miR-181b levels we observed (Figure 1K). Accordingly, areas of macrophage infiltration in AAA tissues were associated with marked MMP activity (Online Figure IV). Furthermore, using the angiotensin II infusion model of AAA in 8-week-old high-fat–fed apolipoprotein E–deficient (*Apoe*^−^^/−^) mice, we investigated macrophage TIMP-3 expression within intact and ruptured abdominal aortas. As in human tissues, significantly more TIMP-3–positive macrophages (3-fold; *P*=0.00315) were present in intact abdominal aortic tissues compared with ruptured aneurysmal samples (Online Figure V). Taken together, our results imply that miR-181b is a critical regulator of macrophage TIMP-3 expression during the progression of atherosclerosis and aortic aneurysms.

### MiR-181b Inhibition Stabilizes Atherosclerotic Plaques in Hypercholesterolemic *Apoe*^−/^^−^ Mice

**Figure 2.**
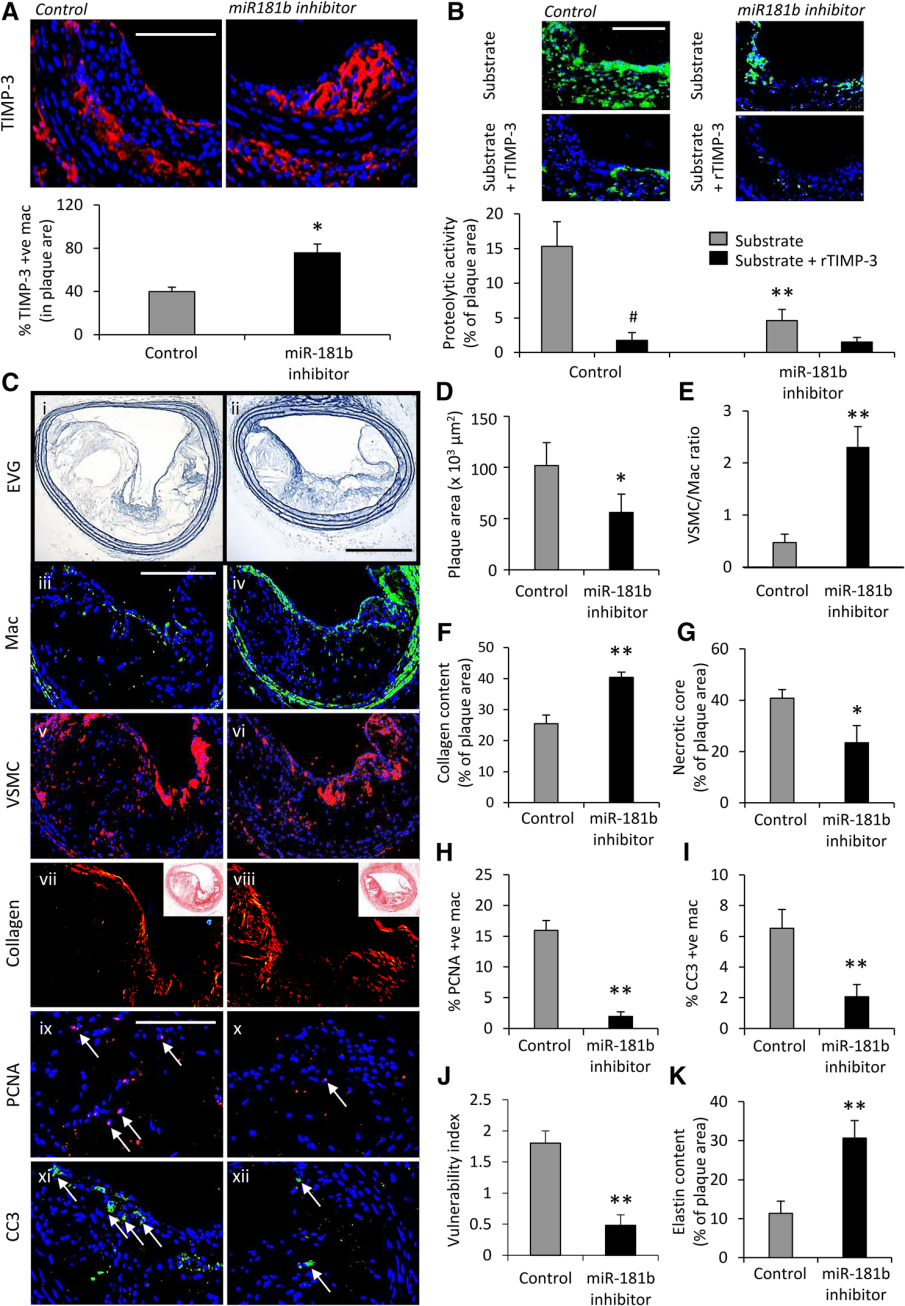
**MicroRNA (miR)-181b inhibition stabilizes atherosclerotic plaques in hypercholesterolemic *Apoe*^−/−^ mice. A**, Representative images and quantification of macrophage tissue inhibitor of metalloproteinase (TIMP)-3 expression as assessed by immunofluorescence staining of brachiocephalic artery plaques from scrambled control and miR-181b inhibitor-treated *Apoe*^−/−^ mice, n=6 to 8/group, *P<0.05, 2-tailed Student *t* test, scale bar represents 50 μm and is applicable to both panels. **B**, Representative images and quantification of proteolytic activity as assessed by in situ zymography of brachiocephalic plaques from scrambled control and miR-181b inhibitor-treated *Apoe*^−/−^ mice, incubated with substrate alone or plus 10 nmol/L recombinant TIMP-3, #*P*<0.05 and represents significant difference from substrate alone; n=6 to 8 per group, **P<0.01 and denotes significant difference from scrambled control mice, ANOVA, scale bar represents 50 μm and is applicable to all panels. **C**, Representative images and quantification of (**D**) plaque cross-sectional area in elastin van Gieson (EVG)–stained sections, (**E**) ratio of total lesional vascular smooth muscle cells (VSMC) and macrophages (Mac) assessed by immunohistochemistry, (**F**) lesional collagen content assessed by picrosirius red staining, (**G**) lesional necrotic core area, (**H**) lesional proliferation percentage determined by immunohistochemistry for proliferating cell nuclear antigen (PCNA), (**I**) lesional apoptosis percentage determined by immunohistochemistry for cleaved caspase-3 (CC3), (**J**) the plaque vulnerability index (necrotic core area+macrophage content/VSMC+collagen content), (**K**) lesional elastin content assessed by EVG staining, in brachiocephalic plaques from scrambled control and miR-181b inhibitor-treated *Apoe*^−/−^ mice, n=6 to 8/group, *P<0.05 and **P<0.01 compared with scrambled control mice, 2-tailed Student *t* test, scale bar in ii represents 100 μm and is applicable to panels i and ii, scale bar in iii represents 100 μm and is applicable to panels iii–viii, scale bar in ix represents 50 μm and is applicable to panels ix–xii. Arrows in panel’s ix–xii indicate positive cells. In all cases, data represent the mean±SEM.

Given the above, we hypothesized that miR-181b inhibition may restore macrophage TIMP-3 expression and prevent the progression of atherosclerosis. Therefore, mice with preexisting atherosclerotic lesions within their brachiocephalic arteries were treated with a locked nucleic acid–modified miR-181b inhibitor or a scrambled miR to serve as a control (n=6–8 per group; see Online Figure VI). Body weights were comparable between scrambled control (29.7±1.1 g) and miR-181b inhibitor-treated mice (30.2±1.3 g), indicating that locked nucleic acid–miR treatment was well tolerated, and no significant effect on lipid profiles was observed (Online Figure VII). Quantitative polymerase chain reaction analysis of atherosclerotic vessels demonstrated reduced miR-181b expression in mice treated with the locked nucleic acid–miR-181b inhibitor controls (Online Figure VIII), inferring that the miR-181b inhibitor had pervaded the plaque/vessel wall. As expected, miR-181b inhibition resulted in a significant increase in intraplaque TIMP-3–positive macrophages (by 90%; *P*<0.05; Figure 2A). As expected, proteolytic activity was abrogated within plaques from miR-181b inhibitor-treated mice when compared with controls (by 70%; *P*<0.01; Figure 2B), as ascertained by in situ zymography, and comparable with the inhibitory effect achieved by addition of exogenous TIMP-3 (Figure 2B). Furthermore, recombinant TIMP-3 displayed no additive inhibitory effect on proteolytic activity in plaques from miR-181b inhibitor-treated animals (Figure 2B), indicating that the miR-181b inhibition-associated increase in TIMP-3 expression was responsible for the diminished proteolytic activity observed in plaques from treated mice. Hence, the TIMP-3–dependent reduction in proteolytic activity afforded through miR-181b inhibition translated into a retardation of plaque progression when compared with control animals, as observed by a reduction in lesion area (by 45%; *P*<0.05; Figure 2C and 2D).

In addition, pathological characteristics associated with a more stable plaque phenotype were increased in treated mice compared with control animals; smooth muscle cell to macrophage ratio (4.9-fold increase; *P*<0.01; Figure 2C and 2E), collagen content (59% increase; *P*<0.01; Figure 2C and 2F), and necrotic/lipid core size (42% decrease; *P*<0.05; Figure 2C and 2G). Moreover, and in line with our previous in vitro data,^8^ intraplaque macrophage proliferation rates and apoptotic frequencies were reduced (88% and 68%, respectively; *P*<0.01; Figure 2C, 2H, and 2I) in brachiocephalic plaques from miR-181b inhibitor-treated mice compared with scrambled control animals. Collectively, miR-181b inhibition resulted in alterations in plaque composition that have been previously taken as markers of increased plaque stability. Consistent with this, the lesion compositional changes translated to a decreased plaque vulnerability index^19^ in mice receiving miR-181b inhibition compared with scrambled control animals (by 73%; *P*<0.01; Figure 2C and 2J). Finally, miR-181b inhibition significantly augmented elastin content within plaques compared with scrambled control animals (2.6-fold; *P*<0.01; Figure 2C and 2K). Collectively, these results suggest that inhibition of miR-181b dramatically increases macrophage TIMP-3 expression and thus retards plaque progression and promotes a more stable phenotype.

We also subjected 10-week high-fat–fed low-density lipoprotein receptor knockout mice (*Ldlr*^−/−^), which have preexisting atherosclerotic lesions within their brachiocephalic arteries (Online Figure IX) to 4-week treatment with the locked nucleic acid–modified miR-181b inhibitor or a scrambled miR to serve as control animals, while being maintained on a high-fat diet (n=6–8 per group, see Online Figure IV). Similar to *Apoe*^−/−^ mice, we observed a significant suppression in plaque progression as observed by a reduction in lesion area of miR-181b inhibitor-treated mice versus controls (by 67%; *P*<0.05; Online Figure IV), indicating that this effect is not exclusive to the *Apoe*^−/^^−^ mouse model.

### MiR-181b Inhibition Does Not Affect Plaque Progression in the Absence of TIMP-3

**Figure 3.**
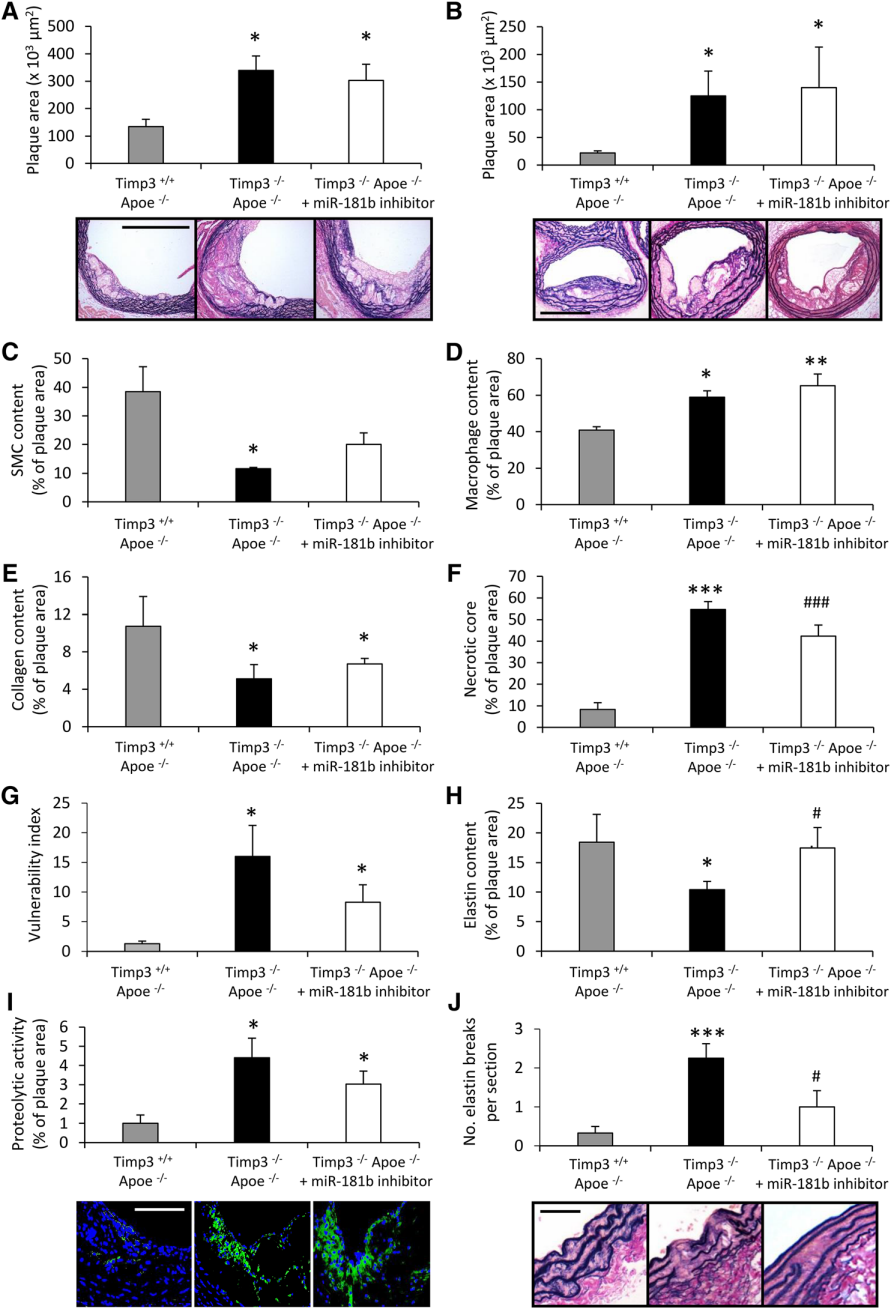
**MicroRNA (miR)-181b inhibition does not affect plaque progression in the absence of tissue inhibitor of metalloproteinase (TIMP)-3**. Representative images and quantification of plaque cross-sectional area in elastin van Gieson (EVG)–stained sections of plaques within (**A**) the aortic root or (**B**) the brachiocephalic artery of *Timp3*^+/+^ *Apoe*^−/−^, *Timp3*^−/−^ *Apoe*^−/−^, and miR-181b inhibitor-treated *Timp3*^−/−^ *Apoe*^−/−^ mice, n=6 to 8/group, *P<0.05 and **P<0.01 compared with *Timp3*^+/+^ *Apoe*^−/−^ control animals, ANOVA, scale bar represents 100 μm and is applicable to all panels. Quantification of (**C**) smooth muscle cell (SMC), (**D**) macrophage, (**E**) collagen content, (**F**) necrotic core area, (**G**) plaque vulnerability index (necrotic core area+macrophage content/vascular smooth muscle cell+collagen content), and (**H**) elastin content, in brachiocephalic plaques from *Timp3*^+/+^ *Apoe*^−/−^, *Timp3*^−/−^ *Apoe*^−/−^, and miR-181b inhibitor-treated *Timp3*^−/−^ *Apoe*^−/−^ mice, n=6 to 8/group, *P<0.05, ***P=0.00013, and ###*P*=0.00938 compared with *Timp3*^+/+^ *Apoe*^−/−^ control animals and #*P*<0.05 compared with *Timp3*^−/−^ *Apoe*^−/−^ mice, ANOVA. **I**, Representative images and quantification of proteolytic activity as assessed by in situ zymography of brachiocephalic plaques from *Timp3*^+/+^ *Apoe*^−/−^, *Timp3*^−/−^ *Apoe*^−/−^, and miR-181b inhibitor-treated *Timp3*^−/−^ *Apoe*^−/−^ mice, n=6 to 8/group, *P<0.05 compared with *Timp3*^+/+^ *Apoe*^−/−^ control animals, ANOVA, scale bar represents 50 μm and is applicable to all panels. **J**, Representative images and quantification of elastin breaks assessed by EVG staining of brachiocephalic plaques from *Timp3*^+/+^ *Apoe*^−/−^, *Timp3*^−/−^ *Apoe*^−/−^, and miR-181b inhibitor-treated *Timp3*^−/−^ *Apoe*^−/−^ mice, n=6 to 8/group, ***P=0.0010 compared with *Timp3*^+/+^ *Apoe*^−/−^ control animals and #*P*<0.05 compared with *Timp3*^−/−^ *Apoe*^−/−^ mice, ANOVA. Scale bar represents 25 μm and is applicable to all panels. In all cases, data represent the mean±SEM.

To assess whether miR-181b inhibition modulates atherosclerotic plaque progression through TIMP-3, we measured plaque development in *Apoe/Timp3* double knockout (*Timp3*^−^^/−^/*Apoe*^−^^/−^) mice and whether miR-181b inhibition retarded the progression of preexisting lesions, as observed in *Apoe* knockout mice. After 12 weeks of high-fat feeding and as expected, *Timp3*^−/−^/*Apoe*^−^^/−^ mice displayed accelerated atherosclerosis compared with control *Timp3*^+/+^/*Apoe*^−^^/−^ animals, as assessed by morphometric analyses of lesion area in cross-sections of the aortic root (2.5-fold; *P*<0.05; Figure 3A) and the brachiocephalic artery (5.7-fold; *P*<0.05; Figure 3B). In contrast to *Apoe*^−^^/−^ mice (Figure 2D), miR-181b inhibition failed to retard plaque progression at either vascular site, in *Timp3*^−^^/−^/*Apoe*^−^^/−^ mice (Figure 3A and 3B). Characterization of brachiocephalic artery atherosclerotic plaques from *Timp3*^−^^/^^−^/*Apoe*^−^^/−^ mice revealed that they contained fewer smooth muscle cells (by 70%; *P*<0.05), but increased macrophage content (by 44%; *P*<0.05), than in plaques from *Timp3*^+/+^/*Apoe*^−^^/−^ control mice (Figure 3C and 3D). Furthermore, whereas there was a reduction in plaque collagen content (by 52%; *P*<0.05), necrotic core area was markedly increased (6.6-fold; *P*<0.01) in plaques from *Timp3*^−^^/−^/*Apoe*^−^^/−^ mice compared with those from control animals (Figure 3E and 3F). Collectively, these findings indicate that pathological markers indicative of increased plaque instability in humans are prominent in *Timp3*^−^^/−^/*Apoe*^−/−^ mice plaques. Indeed, assessment of the vulnerability index showed that this indicator of instability is significantly greater in *Timp3*^−^^/−^/*Apoe*^−^^/−^ mice (12-fold; *P*<0.05) compared with *Timp3*^+/+^/*Apoe*^−^^/−^ control mice (Figure 3G). Consistent with a lack of effect on plaque area, miR-181b inhibition failed to modulate plaque components, such as smooth muscle cell, macrophage, and collagen content, or necrotic core size in *Timp3*^−^^/−^/*Apoe*^−^^/−^ mice (Figure 3), in direct contrast to the beneficial effects observed in miR-181b inhibitor-treated *Apoe*^−/−^ mice (Figure 2). Accordingly, the vulnerability index was unaffected in *Timp3*^−/−^/*Apoe*^−/−^ mice by miR-181b inhibition (Figure 3G). Surprisingly, although plaque elastin content was, as expected, decreased in *Timp3*^−/−^/*Apoe*^−/−^ mice (by 43%; *P*<0.05) compared with *Timp3*^+/+^/*Apoe*^−/−^ control mice (Figure 3H), elastin content was restored to levels comparable with control animals, by miR-181b inhibitor treatment of *Timp3*^−/−^/*Apoe*^−/−^ mice (Figure 3H). In situ zymography demonstrated that plaque proteolytic activity was significantly increased in *Timp3*^−/−^/*Apoe*^−/−^ mice (4.4-fold; *P*<0.05) compared with controls and was unaffected by miR-181b inhibition (Figure 3I), implying that the effect of miR-181b inhibition on plaque elastin was, in part, independent of altered proteolysis. Moreover, whereas it was observed that elastin fragmentation was more prevalent within brachiocephalic arteries from *Timp3*^−/−^/*Apoe*^−/−^ mice (6.8-fold; *P*<0.0010) compared with *Timp3*^+/+^/*Apoe*^−/−^ control mice (Figure 3J), miR-181b inhibition reduced the number of elastin breaks in *Timp3*^−/−^/*Apoe*^−/−^ mice (by 66%; *P*<0.05), although still significantly greater in number than *Timp3*^+/+^/*Apoe*^−/−^ control mice (Figure 3J). Together, these data indicate that the majority of the beneficial actions of miR-181b inhibition on existing atherosclerotic plaques are through restoring macrophage TIMP-3 expression, as most effects were abolished in mice with *Timp3* deficiency. However, modulation of plaque elastin content and fragmentation suggest TIMP-3–independent effects of miR-181b inhibition, implying that miR-181b may regulate other targets during atherosclerosis that influence elastin content. Thus, miR-181b inhibition may have a protective role in other vascular pathologies, particularly aneurysms.

### MiR Inhibition Stabilizes AAAs in Angiotensin II–Infused *Apoe*^−/−^ Mice

**Figure 4.**
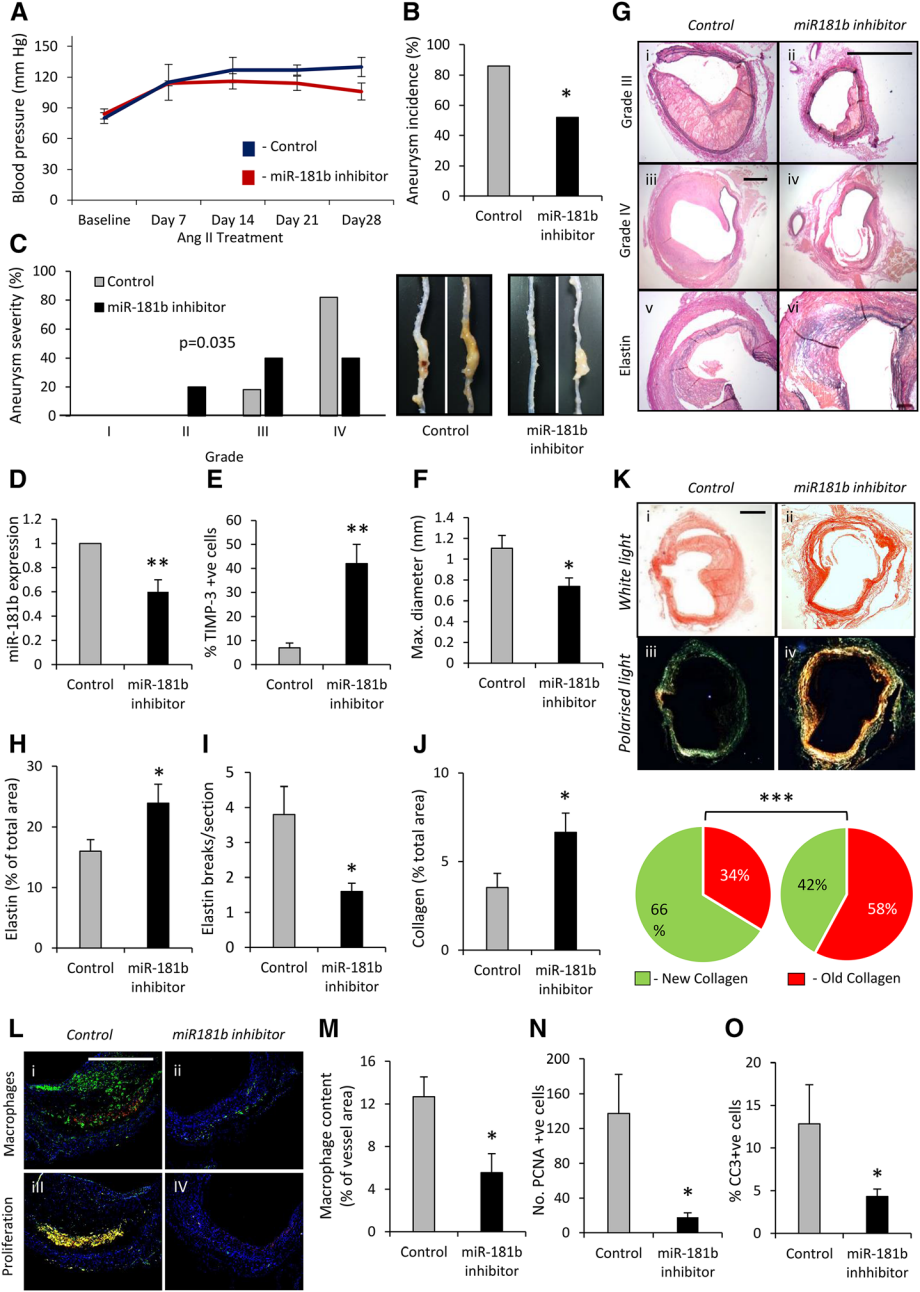
**MicroRNA (miR) inhibition stabilizes abdominal aortic aneurysms (AAAs) in angiotensin II-infused *Apoe*^−/−^ mice. A**, miR-181b inhibition did not alter blood pressure levels. **B**, Quantification of aneurysm incidence and (**C**) severity (increasing severity from stage I to stage IV as described by Raffort et al^12^) in both groups of mice, using Fisher exact test and 2-tailed Student *t* test, respectively, n=6 to 8/group, *P<0.05. **D**, Quantification of miR-181b expression by quantitative polymerase chain reaction (Q-PCR) and (**E**) tissue inhibitor of metalloproteinase (TIMP)-3 protein expression by immunohistochemistry, n=6 to 8/group, **P<0.01 compared with scrambled control mice, 2-tailed Student *t* test. **F**, Maximal abdominal aortic diameter (mm) within the indicated groups, n=6 to 8/group, **P<0.05 compared with scrambled control mice, 2-tailed Student *t* test. **G**, Representative images of elastin van Gieson–stained histological cross-sections of AAAs from scrambled control and miR-181b inhibitor-treated *Apoe*^−/−^ mice, demonstrating the differences in vessel diameter and elastin content (black), scale bar in ii represents 100 μm and is applicable to panels i, ii, and iv–x. Scale bar in iii represents 200 μm and is applicable to panels iii and iv. **H**, Quantification of elastin content and (**I**) elastin breaks in elastin van Gieson–stained AAAs, n=6 to 8/group, *P<0.05 compared with scrambled control mice, 2-tailed Student *t* test. **J**, Quantification of total collagen content in AAAs assessed by picrosirius red staining, n=6 to 8/group, *P<0.05 compared with scrambled control mice, 2-tailed Student *t* test. **K**, Representative picrosirius red staining viewed under white light and linearly polarized light to show fibrillar collagen in AAAs of scrambled control and miR-181b inhibitor-treated *Apoe*^−/−^ mice (scale bar in i represents 200 μm and is applicable to all panels), and associated qualitative analysis of new (green) and old (red) fibrillar collagen fiber content, n=6 to 8/group, *P<0.001 compared with scrambled control mice, Fisher exact test. **L**, Representative images and quantification of (**M**) macrophage content (**N**) proliferation percentage determined by immunohistochemistry for proliferating cell nuclear antigen (PCNA), and (**O**) apoptosis percentage determined by immunohistochemistry for cleaved caspase-3 (CC3), in AAAs from scrambled control and miR-181b inhibitor-treated *Apoe*^−/−^ mice, n=6 to 8/group, *P<0.05 compared with scrambled control mice, 2-tailed Student *t* test, scale bar in i represents 100 μm and is applicable to all panels. In all cases, data represent the mean±SEM.

Using the angiotensin II (Ang II)–induced model of AAA formation in *Apoe*^−/−^ mice fed a high-fat diet,^20^ we investigated the potential beneficial effects of miR-181b inhibition on the progression of infrarenal atherosclerotic AAAs by using the protocol described in Online Figure X. Treatment with an miR-181b inhibitor did not alter mean arterial blood pressure levels in response to Ang II infusion (Figure 4A) but significantly reduced the occurrence of AAAs to 48% from 86% in scrambled inhibitor-infused, control mice (Figure 4B). Other differences noted included the following: decreased AAA severity (Figure 4C), lowered abdominal aortic miR-181b expression by quantitative polymerase chain reaction (40%; Figure 4D), increased TIMP-3 protein expression (Figure 4E), significantly smaller mean maximal abdominal aortic diameters from histology (Figure 4F and 4G), and markedly more elastin (Figure 4G and 4H). Prominent breaks and fragmentation of the elastic lamellae, key features of AAAs, were abrogated in miR-181b inhibitor-treated compared with control animals (Figure 4I) in association with increased collagen accumulation (by 88%; Figure 4J and 4K). By polarimetry, accumulation of red collagen fibers was greater in AAA tissues from miR-181b inhibitor-treated than control mice, indicating thicker and larger collagen fibrils^21^ (Figure 4K). Consistent with the findings in atherosclerotic mice and in line with previous in vitro data showing impaired migration,^8^ macrophage content was diminished in AAAs from miR-181b inhibitor-treated mice compared with scrambled control animals (Figure 4L and 4M), associated with marked suppression of macrophage proliferation rates and apoptotic frequencies (87% and 66% respectively; *P*<0.05; Figure 4L, 4N, and 4O). These results demonstrate that administration of an miR-181b inhibitor augments TIMP-3 expression in AAAs, and this is associated with fewer and more stable aneurysms.

### MiR-181b Inhibition Regulates Matrix Composition at Other Aneurysmal Sites and Is Protective in an Additional Mouse Model

**Figure 5.**
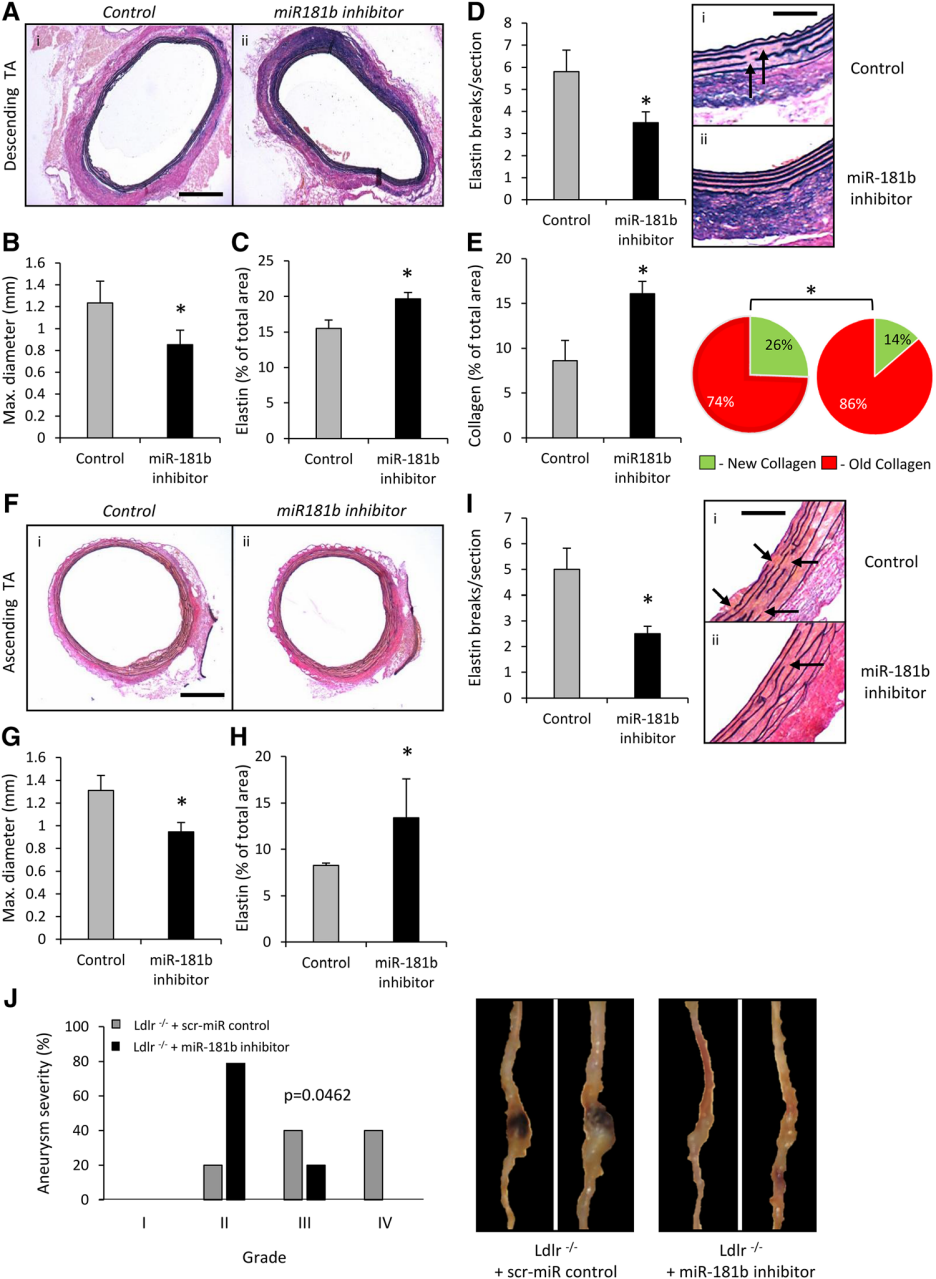
**MicroRNA (miR)-181b inhibition regulates matrix composition at other aneurysmal sites and is protective in an additional mouse model. A**, Representative images and quantification of elastin van Gieson–stained histological cross-sections of descending thoracic aortas (TAs) from scrambled control and miR-181b inhibitor-treated *Apoe*^−/−^ mice demonstrating the differences in (**B**) vessel diameter and (**C**) elastin content (black), n=6 to 8/group, *P<0.05 compared with scrambled control mice, 2-tailed Student *t* test scale bar in i represents 200 μm and is applicable to both panels. **D**, Quantification of elastin breaks in elastin van Gieson–stained descending TAs, n=6 to 8/group, **P<0.01 compared with scrambled control mice, 2-tailed Student *t* test, scale bar in i represents 25 μm and is applicable to both panels, arrows indicate areas of elastin fragmentation. **E**, Quantification of total collagen content and associated qualitative analysis of new (green) and old (red) fibrillar collagen fiber content in descending TAs assessed by picrosirius red staining, n=6 to 8/group, *P<0.05 compared with scrambled control mice, 2-tailed Student *t* test and Fisher exact test, respectively. **F**, Representative images and quantification of elastin van Gieson–stained histological cross-sections of ascending TAs from scrambled control and miR-181b inhibitor-treated *Apoe*^−/−^ mice, demonstrating the differences in (**G**) vessel diameter and (**H**) elastin content (black), n=6 to 8/group, *P<0.05 compared with scrambled control mice, 2-tailed Student *t* test scale bar in i represents 200 μm and is applicable to both panels. **I**, Quantification of elastin breaks in elastin van Gieson–stained ascending TAs, n=6 to 8/group, **P<0.01 compared with scrambled control mice, 2-tailed Student *t* test, scale bar in i represents 25 μm and is applicable to both panels, arrows indicate areas of elastin fragmentation. **J**, Quantification and associated representative images of aneurysm severity (increasing severity from stage I to stage IV as described by Raffort et al^12^) in scrambled control and miR-181b inhibitor-treated *Ldlr*^−/−^ mice, using Fisher exact test, n=6 to 8/group, *P<0.05.

Aortic aneurysms are subdivided anatomically into thoracic aortic aneurysms and AAAs, and although there are some differences in the underlying pathogenesis, both are characterized by fragmented and diminished elastin fibers.^22^ We therefore investigated whether miR-181b inhibition prevents aneurysm formation within the ascending and descending thoracic aortae in our Ang II–infused, *Apoe*^−/−^ mouse model. Mean maximal diameter of descending thoracic aortas in miR-181b inhibitor-treated mice was significantly smaller than those of controls (31%, *P*<0.05; Figure 5A and 5B). The elastin content was also increased together with a decreased number of elastin breaks (Figure 5C and 5D and Online Figure XI). Similar to AAAs, collagen accumulation, particularly more mature collagen fibrils, was increased (Figure 5E). In the ascending thoracic aortas, miR-181b inhibitor-treated mice had decreased vessel expansion compared with controls (by 28%, *P*<0.05; Figure 5F and 5G), which was associated with increased elastin content and reduced elastin fragmentation (Figure 5H and 5I and Online Figure XI). Macrophages were rarely observed at these sites, and TIMP-3 expression was low. Furthermore, no differences in macrophage numbers or TIMP-3 expression were detected between groups (Online Figure XII). These findings show that miR-181b inhibition exerts protective effects on aneurysm formation/progression at multiple susceptible sites within the aorta, even in the absence of overt inflammation, implying additional beneficial effects of miR-181b inhibition independent of increased TIMP-3 protein expression.

To investigate whether the beneficial effects of miR-181b inhibition extended beyond *Apoe*^−/−^ mice, we assessed AAA formation in Ang II–infused, high-fat–fed *Ldlr*^−/−^ mice. AAA severity was significantly reduced in miR-181b inhibitor-treated mice (Figure 5J) compared with scrambled control animals, which exhibited marked aneurysm formation.

### MiR-181b Inhibition Mitigates the Progression of Preexisting AAAs in *Apoe*^−/−^ or *Ldlr*^−/−^ Mice

**Figure 6.**
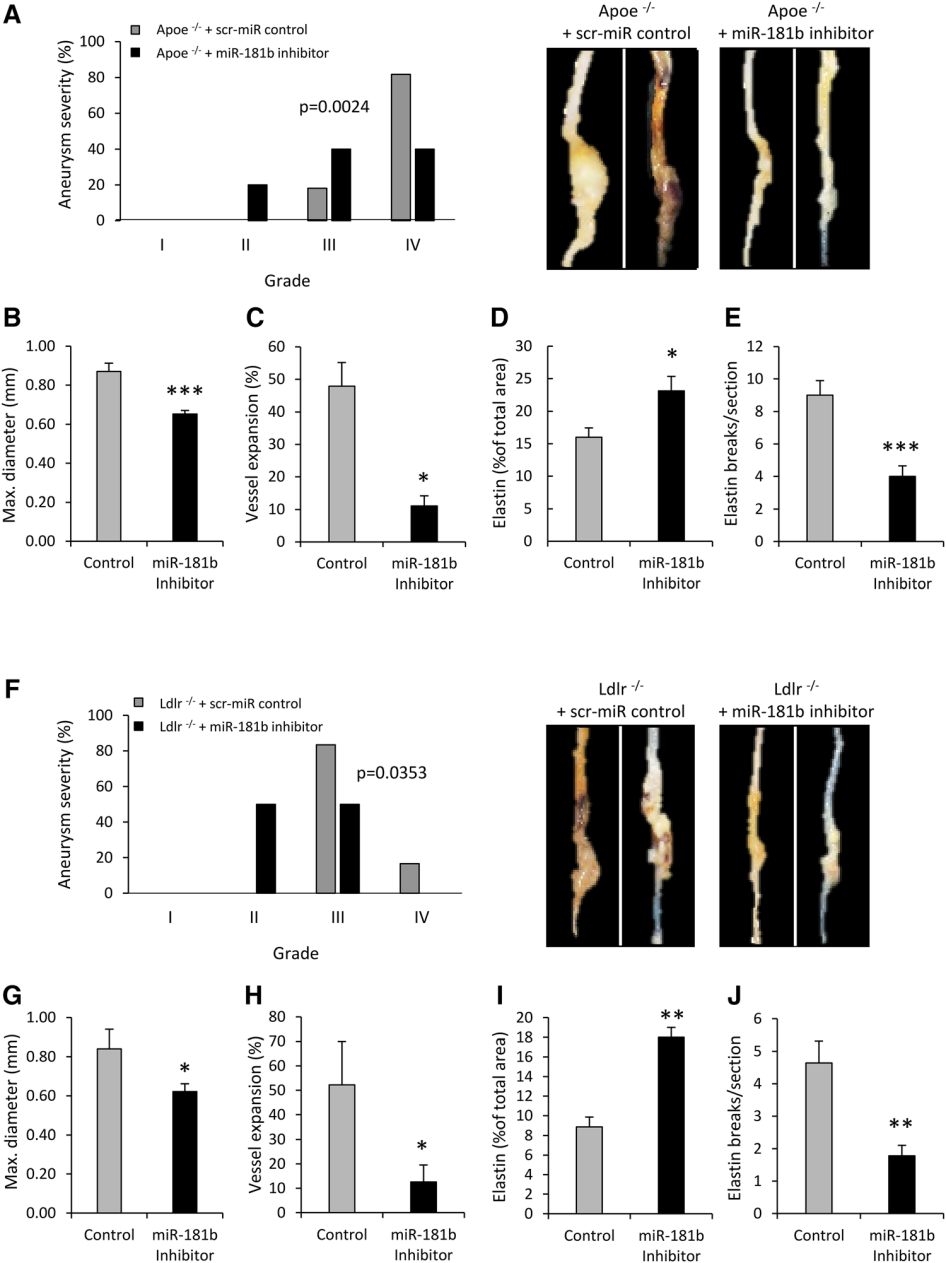
**MicroRNA (miR)-181b inhibition mitigates the progression of preexisting abdominal aortic aneurysms (AAAs) in *Apoe*^−/−^ or *Ldlr*^−/−^ mice. A**, Quantification and associated representative images of aneurysm severity (increasing severity from stage I to stage IV as described by Raffort et al^12^) in scrambled control and miR-181b inhibitor-treated *Apoe*^−/−^ mice with preexisting AAAs, using Fisher exact test, n=6 to 7/group. Quantification of (**B**) vessel diameter, (**C**) vessel expansion, (**D**) elastin content, and (**E**) elastin breaks in scrambled control and miR-181b inhibitor-treated *Apoe*^−/−^ mice with preexisting AAAs, n=6 to 7/group, *P<0.05 and ***P=0.0007 compared with scrambled control mice, 2-tailed Student *t* test. **F**, Quantification and associated representative images of aneurysm severity (increasing severity from stage I to stage IV as described by Raffort et al^12^) in scrambled control and miR-181b inhibitor-treated *Ldlr*^−/−^ mice with preexisting AAAs, using Fisher exact test, n=6 to 7/group. Quantification of (**G**) vessel diameter, (**H**) vessel expansion, (**I**) elastin content, and (**J**) elastin breaks in scrambled control and miR-181b inhibitor-treated *Ldlr*^−/−^ mice with preexisting AAAs, n=6 to 7/group, *P<0.05 and **P<0.01 compared with scrambled control mice, 2-tailed Student *t* test.

To explore the therapeutic potential of miR-181b inhibition, we next investigated its ability to retard the progression of preexisting AAAs. Consequently, hypercholesterolemic male *Apoe*^−/−^ or *Ldlr*^−/−^ mice undergoing Ang II infusion were treated after development of AAAs, according to the protocol shown in Online Figure VA. AAAs from miR-181b inhibitor-treated *Apoe*^−/−^ mice were notably less dilated than those from controls (Figure 6A and 6B). Further expansion in the average baseline maximal diameter before miR-181b inhibition was significantly decreased by miR-181b inhibition compared with scrambled miR control mice (Figure 6C). Moreover, miR-181b inhibitor significantly increased elastin content, as (Figure 6D and Online Figure VI) and reduced the frequency of elastin fragmentation (Figure 6E and Online Figure VI). Similar favorable outcomes were observed in *Ldlr*^−/−^ mice; aneurysm severity, aortic diameter, and associated vessel expansion were all reduced by miR-181b inhibitor treatment (Figure 6F–6H and Online Figure XI). Similarly, the elastin content of AAAs was increased, whereas elastin breaks were reduced relative to controls (Figure 6I and 6J and Online Figure XI). Hence, miR-181b inhibition can also prevent the progression of preexisting AAAs, while increasing the elastin content of advanced AAAs.

### TIMP-3 Protects From Sudden Death Because of Aortic Dissection or Aneurysm Rupture in *Apoe*^−/−^ Mice

**Figure 7.**
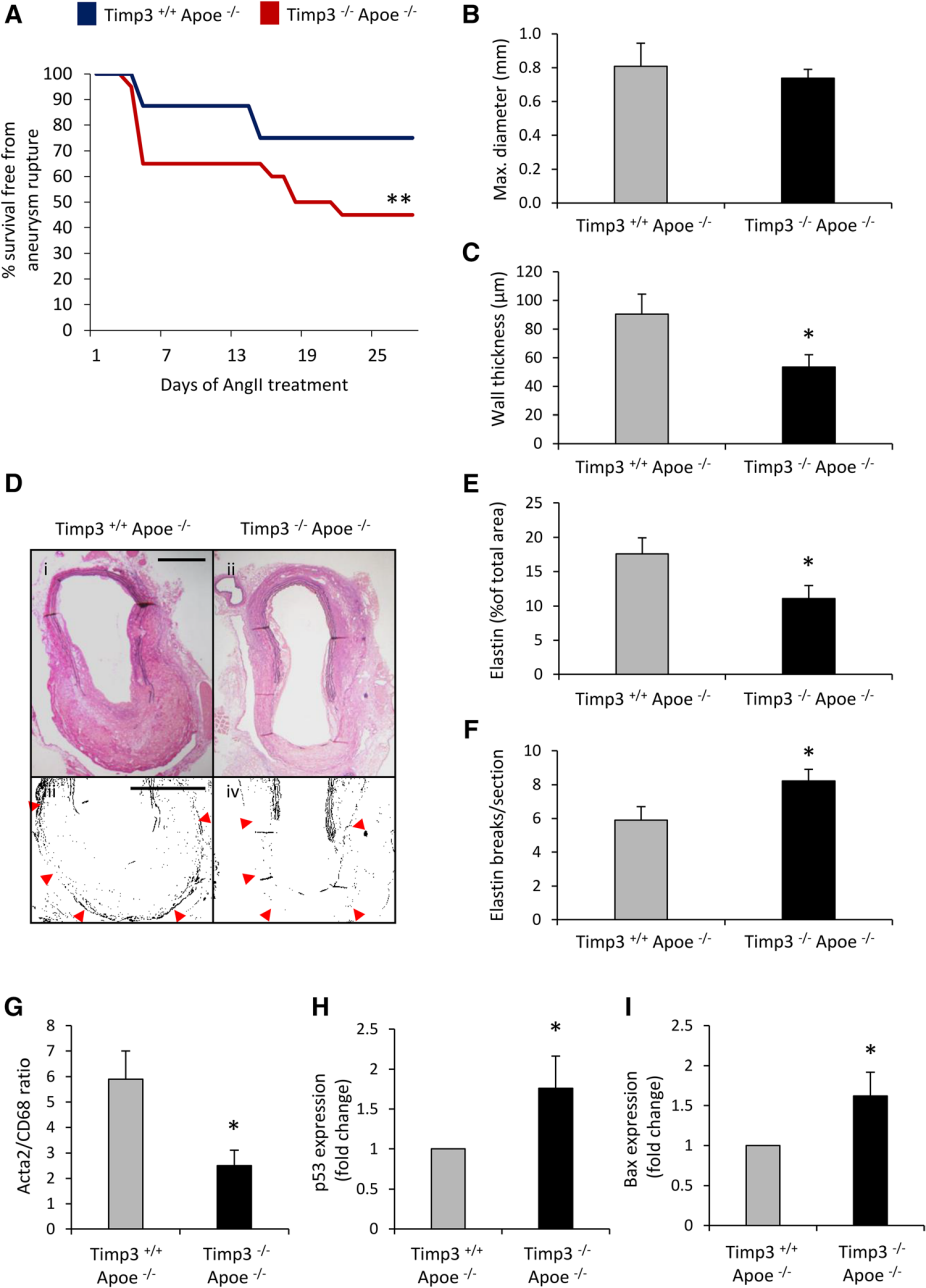
**Tissue inhibitor of metalloproteinase (TIMP)-3 protects from sudden death because of aortic dissection or aneurysm rupture in *Apoe*^−/−^ mice. A**, Kaplan–Meier curves of survival free from aneurysm rupture in 28 days Ang II–infused hypercholesterolemic *Timp3*^+/+^ *Apoe*^−/−^ and *Timp3*^−/−^ *Apoe*^−/−^ mice, n=15 to 20/group. Quantification of (**B**) vessel diameter and (**C**) average wall thickness in *Timp3*^+/+^ *Apoe*^−/−^ and *Timp3*^−/−^ *Apoe*^−/−^ mice, n=6 to 8/group. **D**, Representative elastin van Gieson–stained abdominal aortic aneurysms (AAAs; i and ii) and higher magnification monochrome images (iii and iv) from *Timp3*^+/+^ *Apoe*^−/−^ and *Timp3*^−/−^ *Apoe*^−/−^ mice and associated quantification of elastin content (**E**) and fragmentation (**F**). Scale bars represent 200 μm, and red arrows depict external elastic lamellae. Quantification of (**G**) smooth muscle cell (acta2) to macrophage (CD68) ratio, (**H**) p53, and (**I**) Bax expression in *Timp3*^+/+^ *Apoe*^−/−^ and *Timp3*^−/−^ *Apoe*^−/−^ mice with preexisting AAAs, n=6 to 8/group. Statistical comparisons were made using log-rank test (**A**) or 2-tailed Student *t* test (**B**–**I**), *P<0.05 and **P<0.01 compared with *Timp3*^+/+^ *Apoe*^−/−^ mice. In all cases, data represent the mean±SEM.

To further investigate the role of TIMP-3 in AAA development, *Timp3*^−/−^/*Apoe*^−/−^ and *Timp3*^+/+^/*Apoe*^−/−^ mice were compared in the Ang II–infused high-fat–fed model. Interestingly, whereas 25% of Ang II–infused *Timp3*^+/+^/*Apoe*^−/−^ mice experienced sudden death related to aortic rupture/dissection, the mortality rate of *Timp3*^−/−^/*Apoe*^−/−^ mice was more than doubled (55%, *P*<0.01; Figure 7A). The majority (64%) of *Timp3*^−/−^/*Apoe*^−/−^ mice experienced aortic rupture–related sudden death after 4 to 6 days of Ang II infusion (Figure 7A). Histological examination of AAAs from surviving 28-day Ang II–infused animals revealed that AAA maximal diameter did not differ between groups (Figure 7B), although the average wall thickness was significantly decreased in *Timp3*^−/−^/*Apoe*^−/−^ mice compared with *Timp3*^+/+^/*Apoe*^−/−^ animals (Figure 7C and 7D). *Timp3*^−/−^/*Apoe*^−/−^ mice exhibited decreased elastin content and a greater number of elastin breaks compared with *Timp3*^+/+^/*Apoe*^−/−^ mice (Figure 7D–7F). Moreover, elastin abundance surrounding the aneurysmal sac was less evident in AAAs from *Timp3*^−/−^/*Apoe*^−/−^ mice (Figure 7D). Analysis of AAAs after 7 days Ang II infusion, when rupture and associated death was most prominent in *Timp3*^−/−^/*Apoe*^−/−^ mice, revealed that the ratio of macrophages to smooth muscle cells and markers of apoptosis (p53 and Bax), both characteristics associated with advanced AAAs, were increased relative to *Timp3*^+/+^/*Apoe*^−/−^ mice (Figure 7G–7I). These findings support a critical role for TIMP-3 in AAA rupture that is associated with elastin loss.

### Inhibition of miR-181b Attenuates Mortality Rates in *Timp3*^−/−^/*Apoe*^−/−^ Mice by Directly Stimulating Elastin Expression in VSMCs and AAAs

**Figure 8.**
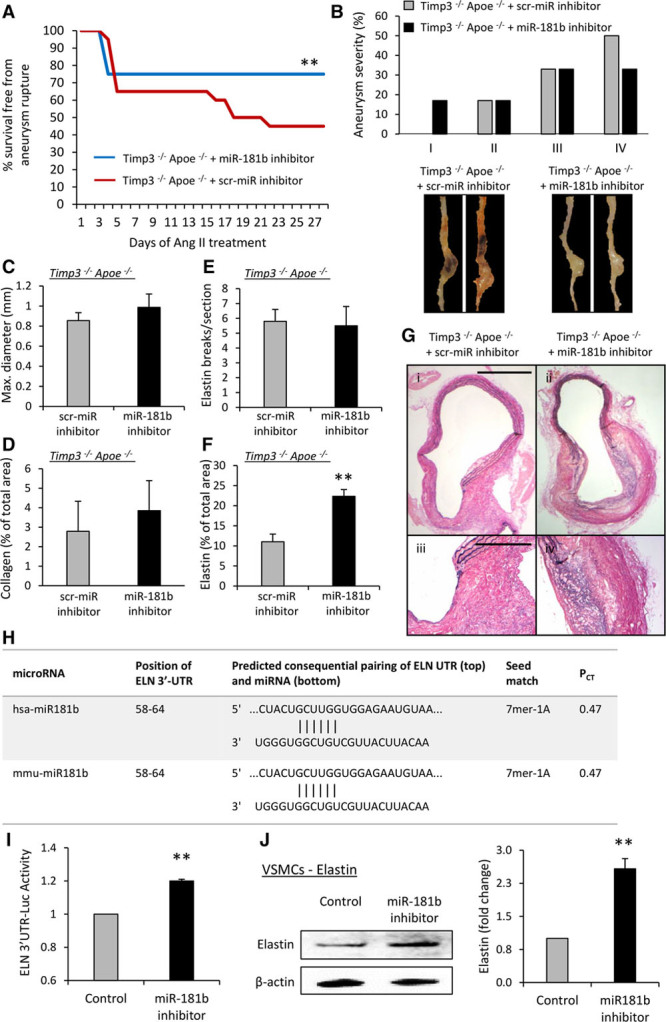
**Inhibition of microRNA (miR)-181b attenuates mortality rates in *Timp3*^−/−^/*Apoe*^−/−^ mice by directly stimulating elastin expression in vascular smooth muscle cells (VSMCs) and abdominal aortic aneurysms (AAAs). A**, Kaplan–Meier curves of survival free from aneurysm rupture in control and miR-181b inhibitor-treated Ang II–infused hypercholesterolemic *Timp3*^−/−^ *Apoe*^−/−^ mice, n=10 to 20/group. **B**, Quantification and associated representative images of aneurysm severity in control and miR-181b inhibitor-treated *Timp3*^−/−^ *Apoe*^−/−^ mice, n=6 to 7/group. Quantification of (**C**) vessel diameter, (**D**) collagen content, (**E**) elastin breaks, (**F**) elastin content, and (**G**) representative images of elastin van Gieson–stained AAAs from control and miR-181b inhibitor-treated *Timp3*^−/−^ *Apoe*^−/−^ mice, n=6 to 7/group, scale bar in i represents 200 μm and is applicable to panels i and ii, scale bar in ii represents 100 μm and is applicable to panels iii and iv. **H**, Conserved miR-181b–binding sites of the 3′-untranslated region (3′-UTR) of human (hsa) and murine (mmu) elastin (*ELN*). P_CT_ refers to the probability of preferentially conserved targeting, demonstrating miR-181b preferentially targets *ELN* in both species. **I**, 3′-UTR luciferase reporter activity of human *ELN* in HeLa cells treated with an miR-181b inhibitor or a scrambled control, n=6. **J**, Representative Western blot and quantification of elastin protein expression in human aortic smooth muscle cells after addition of an miR-181b inhibitor or a scrambled control, n=4. Statistical comparisons were made using log-rank test (**A**), Fisher exact test (**B**), or 2-tailed Student *t* test (**C**–**J**), *P<0.05, **P<0.01, and **P<0.01 compared with controls. In all cases, data represent the mean±SEM.

To test whether miR-181b inhibition protects from AAA progression through TIMP-3, we used *Timp3*^−/−^/*Apoe*^−/−^ mice. Contrary to our expectations, inhibition of miR-181b significantly reduced death rates (from 55% to 25%, *P*<0.01; Figure 8A) after >14 days of Ang II infusion. However, AAA severity and incidence were unaffected (Figure 8B), and no differences in AAA maximal diameter, collagen content, or number of elastin breaks were detected (Figure 8C–8E). Interestingly, elastin content was increased in AAAs of miR-181b inhibitor-treated *Timp3*^−/−^/*Apoe*^−/−^ mice (Figure 8F and 8G), implying that miR-181b modulates elastin expression within AAAs, in part independently from TIMP-3. Using an online database (www.targetscan.org), we identified that mature miR-181b can target both mouse and human elastin mRNA at the 3′-untranslated region (Figure 8H). Indeed, using a wild-type elastin-3′-untranslated region reporter expression vector, we observed that the miR-181b inhibitor increased promoter activity (*P*<0.01; Figure 8I). Considering that vessel wall vascular smooth muscle cells (VSMCs) are the predominant source of elastin production, the effect of miR-181b inhibition on this was assessed. Addition of an miR-181b inhibitor to aortic VSMCs significantly increased elastin protein expression (2.6-fold, *P*<0.01; Figure 8J). Addition of Ang II to VSMCs in culture did not modulate miR-181b expression (Online Figure XIII), implying an indirect effect of Ang II, such as in response to hypertension. Taken together, these findings demonstrate that miR-181b inhibition exerts a dual protective role on AAA progression, through augmenting TIMP-3 expression and directly increasing elastin expression.

## Discussion

The accumulation of macrophages, heightened proteolytic activity, and loss of matrix proteins are considered pivotal events during the progression and rupture of atherosclerotic plaques and aneurysms. We demonstrate here, for the first time, that miR-181b exacerbates these processes and consequently promotes inflammatory cardiovascular diseases. First, we show that miR-181b mediates the downregulation by GM-CSF of macrophage TIMP-3 protein expression. Second, macrophage TIMP-3 protein expression is reduced alongside increased miR-181b levels in both advanced human atherosclerotic plaques and AAAs. Third, miR-181b through TIMP-3 downregulation is a key regulator of numerous macrophage functions involved in plaque and aneurysm progression, including increased MMP activity, macrophage invasion and accumulation, proliferation, and apoptosis. Finally, and most importantly, miR-181b inhibition decreases atherosclerotic plaque formation in mouse models, primarily through upregulation of macrophage TIMP-3 expression, whereas in aneurysm models there is an additional effect on elastin production from VSMC that is supported by in vitro studies. Concomitantly, the composition of atherosclerotic plaques and AAAs is favorably altered and exhibits characteristics associated with stable plaques^23^ and aneurysms^4^ in man. Taken together, these findings imply a dual beneficial effect of miR-181b inhibition during atherosclerosis and AAAs, namely increased macrophage TIMP-3 protein expression and heightened VSMC elastin production, which could eventually be exploited therapeutically.

The progression and rupture of atherosclerotic plaques and AAAs underlie the majority of cardiovascular-related deaths. Elucidating novel pathogenetic factors, such as miR-181b, is therefore paramount for the development of efficient new therapies. Human pathological observations suggest that ECM disruption caused by persistent inflammation drives the formation and progression of atherosclerotic plaques and AAAs, particularly the transition of asymptomatic plaques and small dilatations to clinically relevant plaques and large AAA ruptures.^4,5^ Furthermore, a wealth of mechanistic investigations and studies in relevant animal models have highlighted the critical role of MMPs in collagen and elastin degradation, culminating in atherosclerotic plaque destabilization and medial destruction of the aneurysm wall.^7,17,24–27^ Conversely, TIMPs, by limiting proteolytic activity against ECM and inflammation-related proteins, undoubtedly have protective roles in the progression of atherosclerosis and AAAs.^5^ Although both reduce atherosclerosis and aneurysm formation in mouse models,^28^ TIMP-2 seems to play a greater protective role than TIMP-1 in both atherosclerosis^29^ and aneurysm formation.^30^ The beneficial effect of TIMP-2 is in part through suppression of monocyte/macrophage MMP-14 expression/activity, leading to reduced invasion and accumulation.^29^ MMP-14 (also known as MT1 [membrane type I]-MMP) is inhibited by TIMP-2 and also TIMP-3, but poorly by TIMP-1.^31^ Consistent with this, MMP-14–mediated monocyte/macrophage transmigration across an endothelial monolayer in vitro is efficiently blocked by TIMP-2 and -3, whereas TIMP-1 is ineffective.^32^ TIMP-3 is distinct from TIMP-2 in 2 important aspects; it binds tightly to the ECM, suggesting that it has a principal role in pericellular proteolysis, and it also targets several members of the ADAMs (disintegrin metalloproteinases) family. Previous studies described protective effects of TIMP-3 during atherogenesis and aortic dilatation in rabbits, as well as mice. Atherosclerotic lesion area at the aortic root was increased in *Timp3*^−/−^/*Apoe*^−/−^ mice, alongside heightened inflammation and decreased collagen content.^33^ Conversely, systemic or myeloid cell-specific overexpression of TIMP-3 diminished atherogenesis within an *Apoe*^−/−^ mouse partial carotid artery ligation model^34^ or in the aortic root of *Ldlr*^−/−^ mice.^35^ Deletion of *Timp3* in C57Bl/6 mice infused with Ang II revealed that TIMP-3 reduces adverse remodeling.^36^ Moreover, administration of a broad-spectrum MMP inhibitor rescued vessel enlargement in *Timp3*^−/−^ mice, demonstrating that decreased metalloproteinase activity largely accounted for the protection afforded by TIMP-3.^36^ Our current data and supporting studies demonstrate that expression of TIMP-3 at the mRNA level increases dramatically during monocyte to macrophage differentiation, irrespective of whether this occurs in the presence of M-CSF or GM-CSF.^14^ However, we demonstrated that a distinct subset of foam cell macrophages (which frequent human atherosclerotic plaques and AAAs) exhibit TIMP-3 downregulation (and concomitant increased MMP-14 protein levels).^8^ This unique macrophage phenotype is highly invasive and has increased proliferation and apoptosis rates, all properties expected to destabilize atherosclerotic plaques and AAAs.^8^ The aim of the present study was to elucidate the basis for post-translational regulation of TIMP-3 and investigate its impact during the evolution of inflammation-associated atherosclerosis and AAAs, studies that led to the identification and characterization of miR181b.

In addition to arterial dilatation, AAAs are characterized by decreased medial elastin content and disruption or fragmentation of elastic lamellae,^4^ and MMPs, especially MMP-12, clearly play an important role in this context.^37–40^ Indeed, elastin preservation in the CaCl_2_ experimental model was observed in aortas of select *Mmp*-knockout mice^38,41^ and in mice treated systemically with a c-Jun N-terminal kinase inhibitor to hinder MMP production.^42^ Moreover, numerous studies have evaluated the effects of nonspecific MMP inhibitors in organ culture experiments or animal models of AAAs. Animal studies with doxycycline, a tetracycline analogue that reduces the expression and activity of several MMPs, attenuated elastin fragmentation and loss in rodent AAA models.^43–45^ Similarly, hydroxamate-based broad-spectrum MMP inhibitors also suppressed elastin degeneration within experimental AAAs in murine models.^46–48^ Furthermore, manipulation of individual TIMPs has also demonstrated the ability of these endogenous inhibitors to preserve elastin within the aneurysmal wall.^30,36,49,50^ TIMP-3 augmentation achieved through miR-181b inhibition undoubtedly suppresses the activity of MMPs that target elastin, including MMP-12. We demonstrate here that elastin stabilization is also achieved through a direct effect of miR-181b on elastin protein synthesis. Furthermore, increased elastin content associated with miR-181b inhibition was accompanied by a more stable composition of atherosclerotic plaques and aneurysms, including greater collagen accumulation and enhanced smooth muscle cell to macrophage ratio. Similarly, stabilization and preservation of aortic elastin in the CaCl_2_ AAA model with pentagalloyl glucose prevented aortic dilatation during both development and progression.^51^ Impairment of elastin structure through heterozygous or homozygous mutation of the fibrillin-1 (*Fbn1*) gene is also associated with accelerated atherosclerosis^52^ and aneurysm rupture.^53^ Malformation of mature elastin fibers through fibulin-4 deficiency also results in aortic aneurysms.^54^ The above studies alongside our own findings show that elastin preservation is consistently associated with retardation of aneurysm and atherosclerosis progression. This indicates that elastin stabilization by either TIMP-3–directed MMP inhibition or increased elastin synthesis is both afforded by miR-181b inhibition.

Our previous findings^8^ and current results imply that regulation of both MMP-14 and TIMP-3 expression is both regulated by post-transcriptional mechanisms and that both are under the control of GM-CSF. GM-CSF increases macrophage MMP-14 levels and activity by suppressing miR-24. Moreover, reduction of miR-24 drives advanced atherosclerotic plaque progression.^10^ We show here that GM-CSF sustains miR-181b expression during monocyte-to-macrophage differentiation, and TIMP-3 is therefore inhibited.^10^ GM-CSF also increases MMP-12 expression in macrophages, although this occurs at the level of transcription.^55^ GM-CSF expression is increased in unstable atherosclerotic plaques,^10,56^ and administration of GM-CSF to high-fat–fed *Apoe*^−/−^ mice accelerates atherogenesis.^57^ Similarly, GM-CSF is abundant in human and mouse aortic aneurysms.^58^ Furthermore, GM-CSF administration induced AAA development^57^ and caused aortic dissection^59^ in high-fat–fed *Apoe*^−/−^ mice and CaCl_2_ application/Ang II–infused C57Bl/6 mice, respectively. GM-CSF neutralization abrogated inflammatory aneurysm development and matrix-degrading activity in a *Smad3*^−/−^ mouse model.^58^ Clinically, a positive correlation between plasma concentrations of GM-CSF and intracranial aneurysms has been reported,^60^ and circulating levels of GM-CSF are also elevated in patients with acute aortic dissection.^59^ All of this evidence supports the concept that GM-CSF plays a major part in atherosclerosis and AAAs, in part by sustaining miR-181b levels. In future experiments beyond the present scope, it will be valuable to investigate these concepts further by manipulating the expression of GM-CSF.

We are aware that miR-181b has many additional predicted targets, which may be involved in the advantageous antiatherosclerotic and antianeurysm effects observed in vivo. However, our in vitro, in vivo, and human pathological experiments demonstrate a dominant role of TIMP-3 in protecting from disease progression subsequent to miR-181b inhibition. It has recently been reported that miR-181b can regulate nuclear factor-κB–mediated activation of endothelial cells and ensuing vascular inflammation.^61^ However, effects on atherosclerosis and aneurysm were not assessed, although the authors did demonstrate that miR-181b overexpression in endothelial cells dramatically suppressed TIMP-3 expression.^61^ It is also plausible that other miRs can regulate TIMP-3 expression in atherosclerosis and aneurysms, affecting disease development. Indeed, inhibition of miR-712 (or its human homologue miR-205) prevented endothelial inflammation and atherosclerosis in a carotid ligation model^34^ and aortic dilatation, elastin fragmentation, and aortic rupture in *Apoe*^−/−^ mice,^62^ potentially through post-transcriptional regulation of TIMP-3 and associated heightened MMP activity. In concert with our previous findings^8–10^ and the in situ zymography in the present study, we predict that the activity of select MMPs, such as MMP-14, is retarded through miR-181b–dependent TIMP-3 upregulation, although TIMP-3 can inhibit the activity of multiple MMPs, ADAMs, and aggrecanases (ADAMTS-4 and -5), the individual roles/activities of which were not determined in the current study. Nonetheless, our current findings demonstrate that restoration of TIMP-3 levels achieved through miR-181b inhibition retards the progression of atherosclerotic plaques and aneurysms at multiple vascular beds and in different mouse strains. Furthermore, considering that TIMP-3 has been validated as a target of miR-181b,^13^ our experiments conducted in *Timp3*–deficient mice strongly imply that the beneficial effects afforded by miR-181b inhibition are largely TIMP-3 dependent during atherosclerosis in *Apoe*^−/−^ mice, although an additional protective effect is achieved through elevating elastin synthesis during formation of AAAs.

A further limitation of our study is the use of cell markers and plaque characteristics to infer the stability of atherosclerotic lesions, considering the unproven assumptions inherent in such definitions. However, plaque composition defined by the content of VSMCs and collagen compared with macrophage and lipid is still referred to in the literature as delineators of plaque vulnerability.^63^ Moreover, it has recently been suggested that cells other than macrophages express CD68, which we used as a marker of macrophages in the present study, and subsequently, all mentions to macrophages are in fact CD68^+ve^ cells. Finally, to ensure the reliability of our semiquantitative assessment of histological parameters, intra- and interobserver coefficients were nonsignificant demonstrating that the difference between measurements was within the limits of agreement (Online Figure XIV).

We present here novel in vivo findings that miR-181b inhibition reduces the progression of established atherosclerotic plaques and AAAs, mediated by increased expression of TIMP-3 in intraplaque and intra-aneurysm macrophages and elastin expression in VSMC. Inhibition of miR-181b favorably altered the composition of atherosclerotic plaques and AAAs consistent with improved stability. Furthermore, elevated miR-181b expression occurred in human plaques histologically characterized as stable and correlated with decreased macrophage TIMP-3 expression. Similar results were also observed in human AAA samples when compared with nonaneurysmal aortae. Collectively, these findings support the development of clinically applicable strategies to inhibit miR-181b, thereby maintaining or elevating TIMP-3 and elastin expression, and reducing elastin degradation. Such inhibition of miR-181b could serve as a therapeutic approach in reversing the advancement of atherosclerosis and aortic aneurysms and avoiding the associated acute clinical syndromes.

## Sources of Funding

This work was supported by grants from the British Heart Foundation to J. L. Johnson (FS/07/053/24069 and PG/13/48/30341) and support from the National Institute for Health Research Bristol Cardiovascular Biomedical Research Unit.

## Disclosures

None.

## Supplementary Material

## Novelty and Significance

### What Is Known?

- Rupture of atherosclerotic plaques and abdominal aortic aneurysms underlies most cardiovascular-related deaths.
- Progressive accumulation of macrophages contributes to the pathogenesis of atherosclerosis and aneurysms.
- Macrophages at rupture-prone sites of plaques and aneurysms display increased expression and activity of several matrix metalloproteinases but decreased expression of tissue inhibitor of matrix metalloproteinases (TIMP)-3.
