# Supplementary material for: MicroRNA-181b Controls Atherosclerosis and Aneurysms Through Regulation of TIMP-3 and Elastin
Source: Circ Res. 2017 Jan 5;120(1):49–65. doi: 10.1161/CIRCRESAHA.116.309321 (PMC5214094; doi:10.1161/CIRCRESAHA.116.309321)
Supplement: Supplementary file 1 [file res-120-49-s001.pdf]

Supplemental Material

Methods

Human coronary and aortic samples

Coronary artery segments were collected from cadaveric heart donors from the Bristol Valve Bank, and incorporated into the Bristol Coronary Artery Biobank under National Research Ethics Service approval (08/H0107/48). Valve donor patients had died of non-cardiac related pathologies, and from the limited information retrieved under our ethics, were not prescribed statins or other known athero-modifying medications. The average age of patients with histologically defined stable and unstable plaques was 56±2 and 59±2 years, respectively and a 6/4 male to female ratio. Human aneurysmal abdominal aortic samples and non-aneurysmal aortic specimens were collected under National Research Ethics Service approval (11/H0102/3). Q-CPR for miR-181b was conducted and data analysed after normalization to Scarna17 expression and CD68 mRNA expression. Paraffin sections from histologically classified stable or unstable coronary artery plaques alongside aneurysmal and non-aneurysmal aortas, were stained with a CD68 antibody to detect macrophages and a TIMP-3 antibody, and the percentage of TIMP-3 positive macrophages quantified.

In situ hybridisation

For the detection of miR-181b in human stable and unstable coronary atherosclerotic plaques, 3µm serial sections were subjected to in situ hybridisation (ISH). In brief, cleared slides were underwent citrate buffer-induced antigen retrieval and subsequent immersion in 0.2M HCl for 20 minutes. After three washes in 0.3% triton-X, slides were incubated with 15µg proteinase K at 37°C for 20 minutes then fixed with 3% PFA for 10 minutes. Following incubation with hybridisation buffer (50% formamide, 4 x SSC, 2.5 x Denhadrt's solution, 2.5mg/ml salmon DNA, 0.6mg/ml yeast tRNA, 0.025% SDS and 0.1% blocking reagent) at 60°C for 1 hour slides were incubated with 70µl of 40nM 5', 3' double digoxigenin-labeled locked nucleic acid (LNA)-modified miRCURY miR-181b detection probe or scrambled miRCURY LNA oligonucleotide to serve as a negative control (Exiqon, Denmark), in the same buffer at 60°C overnight. After stringency washing with different concentrations of SSC buffer and blocking for 1 hour (1% blocking reagent in PBS and 10% FCS), HRP-conjugated mouse monoclonal antibody to digoxigenin (DIG) (Abcam) diluted in 1x blocking buffer at a concentration of 1:200 was added for 1 hour. After washing (0.1M Tris PH 9.0), AlexaFluor-594-labelled tyramide (Invitrogen) was added to each section and incubated in the dark at room temperature for 1 hour. After washing slides were mounted with ProLong Gold with Dapi (Invitrogen). The number of miR-181b positive macrophages as assessed by CD68 staining on serial sections, was assessed and expressed as percentage of $CD68^+$ cells positive for mir-181b..

Animals

Timp3 $^{-/-}$ mice (FVB/N strain background)were provided by Professor Dylan Edwards (University of East Anglia) (1). Apoe $^{-/-}$ and Ldlr $^{-/-}$ mice (both C57Bl/6N strain background) were purchased from the Jackson Laboratory and maintained within our own animal facility. Apoe $^{-/-}$ mice were crossed with Timp3 $^{-/-}$ mice to generate Timp3 $^{-/-}$/Apoe $^{-/-}$ double knockout mice as well as their relevant age-, strain-, and sex-matched Timp3 $^{+/+}$/Apoe $^{-/-}$ single knockout littermate controls.

Atherosclerosis studies

In order to assess effects on established atherosclerotic lesions within the brachiocephalic arteries of Apoe $^{-/-}$ mice, female animals at the age of 8-10 weeks were fed a high-fat diet containing 21% (wt : wt) pork lard and supplemented with 0.15% (wt : wt) cholesterol (Special Diet Services, Witham, UK) for 12 weeks, as previously demonstrated (2). Due to Ldlr $^{-/-}$ mice exhibiting milder total cholesterol levels and associated slower plaque development than their Apoe $^{-/-}$ counterparts (3), 8-10 week old male Ldlr $^{-/-}$ mice were high-fat fed for 14 weeks to induce the formation of intermediate plaques. The above experimental plans are illustrated in Supplementary Figure VI.

Mouse model of Ang II–induced aortic aneurysm

To induce AAA formation in atherosclerotic plaque-prone Apoe $^{-/-}$ or Ldlr $^{-/-}$ mice, male animals at the age of 8-10 weeks were fed a high-fat diet containing 21% (wt : wt) pork lard and supplemented with 0.15% (wt : wt) cholesterol (Special Diet Services, Witham, UK) for 8 weeks, the last four weeks of which they were infused with Ang II as previously described (4). The above experimental plans are illustrated in Supplementary Figure X. Alzet model 1007D, 1002 and 1004 osmotic mini-pumps (Charles River UK) implanted subcutaneously were used to deliver Ang II (500 or 1000 ng/kg/min, Enzo Life Sciences), for 7, 14, or 28 days, depending on the experimental design. Mice were anaesthetised with isoflurane and oxygen (2.5% and 1L/min) which was followed by mini-pump implantation. Post-operative analgesia was performed by intraperitoneal injection of buprenorphine (0.1mg/kg), immediately after surgery and animals closely monitored for 24 hours and subsequent pain relief delivered if necessary. To ensure that the studies were adequately powered, group sizes of at least 10 animals were used per group, accepting a 15-20% attrition rate due to early aortic rupture and sudden death. To assess the incidence and severity of AAA formation, the method outlined by Daugherty and colleagues was adopted (5). Aneurysms were graded based on the following scale: Type I, dilated lumen in the supra-renal region of the aorta with no thrombus. Type II, remodelled tissue in the supra-renal region that frequently contains thrombus. Type III, a pronounced bulbous form of type II that contains thrombus. Type IV, a form in which there are multiple aneurysms containing thrombus, some overlapping, in the suprarenal area of the aorta. Aneurysmal tissue was categorized independently by two blinded observers. There was complete concordance in the designation by the two observers.

In vivo miR-181b inhibition

Atherosclerosis study: 8 mg/kg dose of either a LNA-anti-miR181b or scrambled (scr)-miR (miR-CURY LNA miR inhibitor from Exiqon) was injected via the tail vein of high fat-fed Apoe $^{-/-}$ or Ldlr $^{-/-}$ mice (as previously described (6)). Injection was repeated after 4 days and sample collected 3 days after the second injection. Aneurysm study: 4 mg/kg dose of either a LNA-anti-miR181b or scr-miR was delivered by intraperitoneal injection of high fat-fed Apoe $^{-/-}$ or Ldlr $^{-/-}$ mice at the same time as Ang II delivery commenced (to assess effects on AAA development), or 28 days after Ang II-infusion (to assess effects on AAA progression). In both instances injections were repeated weekly, for four weeks for the AAA development study, and two weeks for the AAA progression study.

Termination

Animals were anaesthetised by intraperitoneal injection of sodium pentobarbitone (500mg/kg), before exsanguination by perfusion via the heart with PBS at a constant pressure of 100 mmHg, with outflow through the incised jugular veins. This was followed by constant pressure perfusion with 10% formalin for at least 5 minutes.

Cholesterol Assay

Cholesterol levels from mouse plasma were quantified using the BioVison Cholesterol Assay Kit (Cambridge BioSciences, Cambridge, UK).

Characterisation of atherosclerotic plaques

Up to four vessel cross-sections were quantified per mouse, between 150 and 200µm apart. Paraffin-embedded brachiocephalic artery sections were used. Elastin staining was visualized using Elastin van Gieson histochemistry for plaque morphometric analysis. Up to five vessel cross-sections were quantified per mouse. Analysis was performed using a computerised image analysis program (Image Pro Plus, DataCell, Maidenhead, UK). The lengths of the internal and external elastic lamellae were recorded by image analysis. These were used to derive the total vessel area and the (lumen + plaque) area, by assuming them to be the circumferences of perfect circles. Plaque area was measured directly, and was subtracted from the area enclosed by the internal elastica to derive the lumen area.

Characterisation of aneurysmal lesions

Up to four vessel cross-sections were quantified per mouse, between 150 and 200µm apart. Paraffin-embedded ascending, descending and abdominal aortic sections were used. Elastin staining was visualized using Elastin van Gieson histochemistry, and the number of degraded elastin lamellae and elastin content was quantified by a researcher blinded to the experimental protocol. Morphometric analysis was performed using a computerised image analysis program (Image Pro Plus, DataCell, Maidenhead, UK). The lengths of the internal and external elastic lamellae were recorded by image analysis. These were used to derive the total vessel area, lumen area, mean wall thickness, and maximum diameter by assuming them to be the circumferences of perfect circles. Plaque area was measured directly, and was subtracted from the area enclosed by the internal elastica to derive the lumen area.

Histological and immunohistochemical analysis

Up to four vessel cross-sections were quantified per mouse, between 150 and 200µm apart. Brachiocephalic artery and aortic sections were cut at 3µm and subjected to the following histo- and immunohisto-chemical analyses. Quantitative analysis of fibrillar collagen content was performed using picrosirius red staining of sections viewed under polarised light. Qualitative analysis of fibre thickness was assessed by delineating green and red fibres disposed under polarised light, as fibre colour variation progresses from green to red proportionally to the increase of fibre thickness/ages, as described previously (7) The relative amount of each fibre colour was expressed as a percentage of the total amount of collagen in the area of interest. Quantitative analysis of elastin content and elastin breaks was performed using elastin van Gieson stained sections (four sections/vessel of interest/animal, 200µm apart). The relative amount of elastin (which appears as black under light microscopy) was determined using a computerised image analysis program (Image Pro Plus, DataCell, Maidenhead, UK) and expressed as an average percentage in the area of interest (plaque or aneurysmal artery), whilst the number of elastin breaks per section were counted and expressed as average number of elastin breaks per section averaged from four sections taken 200µm apart. Sections were subjected to immunohistochemistry for smooth muscle cells (α-smooth muscle actin), macrophages (CD68), TIMP-3, proliferation cell nuclear antigen (PCNA) or cleaved caspase-3 (CC3) as described previously (6). Primary antibody details are listed in Supplemental Table 1. Dual immunohistochemistry was performed by incubating sections with two appropriate primary antibodies simultaneously. Cells stained positive with the cell-specific markers were counted and density expressed as the percentage of total nucleated cells stained positive per plaque, or as percentage of positive area of interest. Vulnerability index was calculated by dividing the percentage of plaque area occupied by macrophages and necrotic core, by that of vascular smooth muscle cells and collagen. A score significantly >1 implies increased vulnerability, conversely significantly <1 suggests heightened stability (8).

In situ zymography

Gelatinolytic activity was localised in arteries removed from *Apoe* knockout mice that had received high-fat diet for 13 weeks treated with a miR-181b inhibitor or scrambled control for the last 7 days, as previously described (6). In brief, frozen 8 µm cryostat sections were incubated overnight at room temperature in a humidified dark chamber with 20 µg/mL DQ™ Gelatin (Life Technologies Ltd, Paisley, UK) dissolved in developing buffer (50 mM Tris, pH 7.4, 150 mM NaCl, 5 mM CaCl2, 0.2 mM sodium azide). Cleavage of the substrate by proteinases results in unblocking of quenched fluorescence and in an increase in fluorescence intensity. Sections were incubated for 24 hours in developing buffer alone, or in the presence of recombinant TIMP-3 (10nM; 973-TM-010; R&D Systems). Sections were washed in PBS, fixed with 4% paraformaldehyde, and mounted with ProLong® Gold antifade reagent with DAPI (Life Technologies Ltd, Paisley, UK). Using fluorescence microscopy, gelatinolytic activity was identified as green fluorescence.

Monocyte Isolation, macrophage maturation, and gene expression analysis

Peripheral blood mononuclear cells were isolated by differential centrifugation from whole blood of healthy donors, which were collected under South West 4 Research Ethics Committee reference 09/H0107/22. Blood (24mL per donor) was diluted with Dulbecco's Phosphate Buffered Saline (PBS) without calcium and magnesium (Lonza) (ratio 1:1). The diluted samples were subjected to density gradient separation on Ficoll Paque Plus (ratio 1:1) (GE Healthcare Life Sciences) and centrifuged. After centrifugation the PBMC layer was collected and washed in Hank's Balanced Salt Sodium (HBSS) with phenol red without calcium and magnesium (Lonza). Monocytes were isolated by adhering the peripheral blood mononuclear cells to tissue culture plastic for 2 hours at a concentration of 2.5×10^6 cells/mL. Monocytes were cultured in RPMI media with 2mM L-glutamine, 100 IU/mL penicillin, 100 µg/mL streptomycin, 10% fetal bovine serum (FBS; Lonza, Sigma), and either 20 ng/mL recombinant human macrophage-colony stimulating factor (M-CSF; R & D systems) or recombinant human granulocyte macrophage-colony stimulating factor (GM-CSF; R & D systems), for 7 days to generate mature macrophages (6). The Qiagen miRNeasy kit was used for total RNA extraction including micro RNA (Qiagen) in accordance with the manufacturer's protocol. Recover-All kit (Ambion) was used for RNA isolation from paraffin-embedded sections according to the manufacturer's instructions. RNA samples were quantified with a NanoDrop ND-1000 spectrophotomter (LabTech International). Equal amounts of cDNA were generated using a miScript Reverse Transcription Kit (Qiagen) Real-time quantitative PCR was performed in a Roche Light Cycler 1.5 to quantify the steady-state concentration of RNA using a QuantiTect SYBR Green PCR Kit and primers as detailed in Supplemental Table II. Samples were incubated first at 42°C for 30 minutes then at 95°C for 3 minutes. The cDNA obtained was stored at -80°C.

Macrophage and vascular smooth muscle cell miR-181b inhibitor transfection
The RVG-9dR peptide (9) was used to deliver anti-has-miR-181b miScript miRNA inhibitor (10nM) or a scrambled control (Qiagen) to 7 day GM-CSF matured macrophages, as described previously (6). Four hours post-transfection in serum free antibiotic free RPMI, the media was changed and fresh RPMI/FCS was added to each sample. Cells were then cultured for 24 hours before collection in SDS lysis buffer. Human aortic vascular smooth muscle cells were cultured in DMEM containing 10% FCS. Cells were transfected with 50nM anti-has-miR-181b miScript miRNA inhibitor (Qiagen) using Lipofectamine RNAiMax (Invitrogen) according to the manufacturer's instructions. Appropriate negative controls were also utilised (0.1 nM All Stars Negative Control (Qiagen). Cells were cultured for at least 24 hours before collection for downstream analysis.

Western Blotting
SDS lysis buffer was used to extract macrophage or VSMC proteins and total protein concentration was measured using a bicinchoninic acid protein assay kit (Pierce). Equal protein concentrations were loaded and electrophoresed on 4-12% gradient gels (Novex Bis-Tris gel) and transferred to 0.2µm nitrocellulose membranes. Blots were blocked with 5% (w/v) skimmed milk powder and incubated overnight at 4^0C with anti-TIMP-3 or anti-elastin antibody (see Supplemental Table I) diluted in SignalBoost™ Solution 1 (Merck). Primary antibodies were detected using species-relevant HRP-conjugated secondary antibodies diluted in SignalBoost™ Solution 2 (Merck), and enhanced Luminata Forte chemiluminescence reagent (Merck). Optical density of bands was quantified using densitometry (Quantity One) and normalised to a β-actin loading control.

Luciferase Reporter Assay
Hela cells were cultured in RPMI-1640 10% FCS. The day before transfection cells were detached with trypsin and seeded at 1.5×10^5 cells per well in 6 well plates. 18 hours post seeding the media was changed to serum/antibiotic-free RPMI 1640 with L-Glutamine. 4 hours after the cells were transfected with Lipofectamine 2000 (Invitrogen) vector. A transfection mix containing Lipofectamine 2000 (1:500 ratio) in serum/antibiotic-free RPMI 1640 with L-Glutamine, 25ng Renilla and 500ng Eln luciferase reporter plasmid were added to all cells. Cells were co-incubated for 4 hours at 37°C, with

anti-has-miR-181b miScript miRNA inhibitor (10nM) or a miScript Inhibitor Negative Control (Qiagen; cat. No. 1027272) termed 'scrambled control', and then RPMI/FCS added to all relevant samples. 24 hours after transfection samples were collected in Dual-luciferase Assay Lysis buffer (Promega). Samples were prepared and quantified in accordance with the kit datasheet.

Study approval

Mice - The housing and care of the animals and all the procedures used in these studies were performed in accordance with the ethical guidelines and regulations of the University of Bristol and the UK Home Office. The investigation conforms to the Guide for the Care and Use of Laboratory Animals published by the US National Institutes of Health (NIH Publication No. 85–23, Eighth Edition, revised 2011). Adherence to the ARRIVE guidelines (10) for the reporting of animal in vivo experiments was also followed.

Human tissues – Coronary artery and aortic tissue segments were collected from cadaveric heart donors to the Bristol Coronary Artery Biobank under National Research Ethics Service approval (08/H0107/48). Written consent was obtained from the next of kin as part of the organ donation programme. Abdominal aortic aneurysm segments were collected from consenting patients undergoing aneurysm repair surgery under National Research Ethics Service approval (11/H0102/3). Consent was not required as the tissue is considered surplus.

Statistical Analysis

Values are expressed as mean ± standard error of the mean (SEM). Group values were compared using the computer program InStat (GraphPad). For the comparison of group means, a check was first made for normal distribution: if this was passed then an unpaired two sample two-tailed Student's t-test was carried out. If the variances were significantly different, then a Welch's correction test was used. Statistical differences between monocyte/macrophages from the same preparation were analysed by Students paired t-test. For the comparison of multiple groups, an analysis of variance (ANOVA) test was used, and a Student–Newman–Keuls multiple comparisons post hoc test employed when statistical differences were detected. Contingency data (for example aneurysm incidence) were analysed by Fisher's exact test. All in vivo and histological analyses were performed by two investigators in a blinded fashion. To demonstrate the robust assessment of semi-quantitative parameters such as plaque macrophage content and positivity for TIMP-3, intra- and inter-observer variability was determined by Bland –Altman plots. In all cases, statistical significance was concluded where the two-tailed probability was less than 0.05.

References

1. Singh RJR, Mason JC, Lidington EA, Edwards DR, Nuttall RK, Khokha R, Knauper V, Murphy G, Gavrilovic J. Cytokine stimulated vascular cell adhesion molecule-1 (VCAM-1) ectodomain release is regulated by TIMP-3. *Cardiovasc Res.* 2005;67:39-49.
2. Johnson JL, Fritsche-Danielson R, Behrendt M, Westin-Eriksson A, Wennbo H, Herslof M, Elebring M, George SJ, McPheat W, Jackson CL. Effect of Broad-Spectrum Matrix Metalloproteinase Inhibition on Atherosclerotic Plaque Stability. *Cardiovasc Res.* 2006;71:586-95.
3. Veniant MM, Withycombe S, Young SG. Lipoprotein size and atherosclerosis susceptibility in Apoe(-/-) and Ldlr(-/-) mice. *Arterioscler Thromb Vasc Biol.* 2001;21:1567-70.
4. Daugherty A, Manning MW, Cassis LA. Angiotensin II promotes atherosclerotic lesions and aneurysms in apolipoprotein E deficient mice. *J Clin Invest.* 2000;105:1605-12.
5. Daugherty A, Manning MW, Cassis LA. Antagonism of AT2 receptors augments Angiotensin II-induced abdominal aortic aneurysms and atherosclerosis. *Br J Pharmacol.* 2001;134:865-70.
6. Di Gregoli K, Jenkins N, Salter R, White S, Newby AC, Johnson JL. MicroRNA-24 Regulates Macrophage Behavior and Retards Atherosclerosis. *Arterioscler Thromb Vasc Biol.* 2014;34:1990-2000.

7. Quillard T, Tesmenitsky Y, Croce K, Travers R, Shvartz E, Koskinas KC, Sukhova GK, Aikawa E, Aikawa M, Libby P. Selective Inhibition of Matrix Metalloproteinase-13 Increases Collagen Content of Established Mouse Atherosclerosis. *Arterioscler Thromb Vasc Biol.* 2011;31:2464-72.
8. Shiomi M, Ito T, Hirouchi Y, Enomoto M. Fibromuscular cap composition is important for the stability of established atherosclerotic plaques in mature WHHL rabbits treated with statins. *Atherosclerosis.* 2001;157:75-84.
9. Kim SS, Ye CT, Kumar P, Chiu I, Subramanya S, Wu HQ, Shankar P, Manjunath N. Targeted Delivery of siRNA to Macrophages for Anti-inflammatory Treatment. *Mol Ther.* 2010;18:993-1001.
10. Kilkenny C, Browne WJ, Cuthill IC, Emerson M, Altman DG. Improving Bioscience Research Reporting: The ARRIVE Guidelines for Reporting Animal Research. *PLoS Biol.* 2010;8:e1000412.

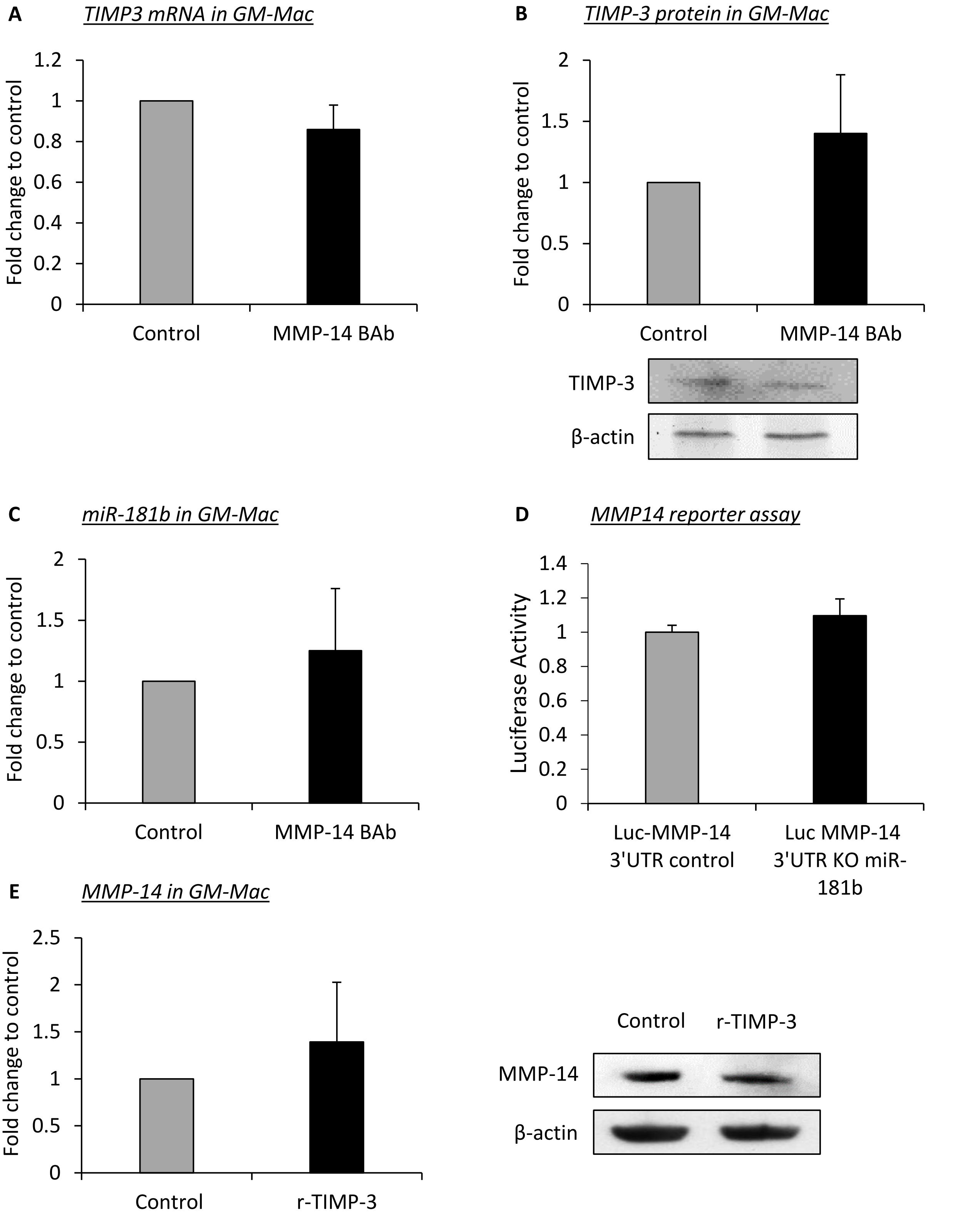

Supplemental Figure I: TIMP-3 and miR-181b expression are not regulated by MMP-14, and MMP-14 is not a miR-181b target gene

(**A**) QPCR and (**B**) western blot of TIMP-3 mRNA and protein expression respectively, in human macrophages differentiated in the presence of GM-CSF and treated with a MMP-14 blocking antibody (BAb; 15ng/ml) or mouse IgG to serve as a control, n=4/group. (**C**) QPCR of miR-181b in human macrophages differentiated in the presence of GM-CSF and treated with a MMP-14 blocking antibody (BAb; 15ng/ml) or mouse IgG to serve as a control, n=4/group. (**D**)) 3'-UTR luciferase reporter activity of human MMP14 in HeLa cells treated with a miR-181b inhibitor or a scrambled control, n=6. (**E**) Western blot of TIMP-3 protein expression in human macrophages differentiated in the presence of GM-CSF and treated with recombinant TIMP-3 (10nM), n=4/group.

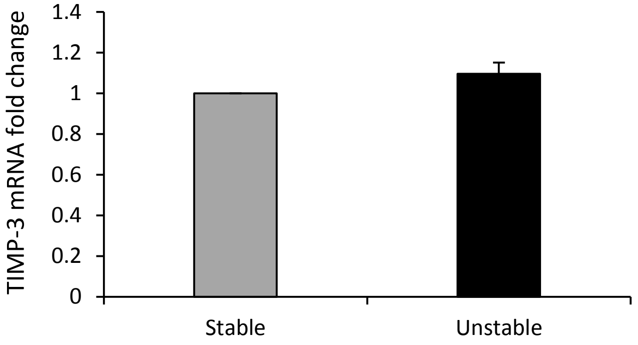

Supplemental Figure II: TIMP-3 mRNA expression is not altered between stable and unstable plaques

QPCR analysis of TIMP-3 mRNA expression from stable and unstable coronary atherosclerotic plaques, n=10/group. Data is expressed as fold change in expression compared to stable plaques, which is normalised to Scarna17 expression and set to 1.

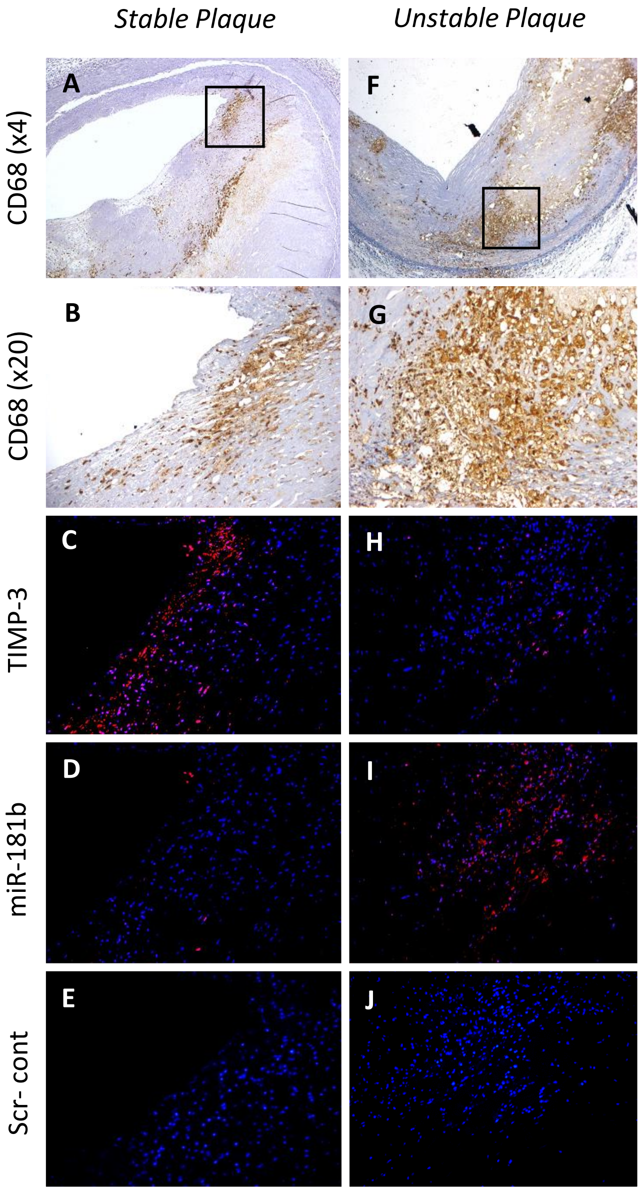

Supplemental Figure III: MicroRNA-181b expression is inversely related to macrophage TIMP-3 expression and plaque stability in human coronary atherosclerosis

Representative images of CD68 (for macrophages) and TIMP-3 protein expression by immunohistochemistry, and miR-181b or a scrambled LNA oligonucleotide to serve as a negative control (Scr-cont) by in situ hybridisation, from stable (A-E) and unstable (F-J) coronary atherosclerotic plaques, n=10/group. Box in A and F, higher magnification in B–E and G–J, respectively.

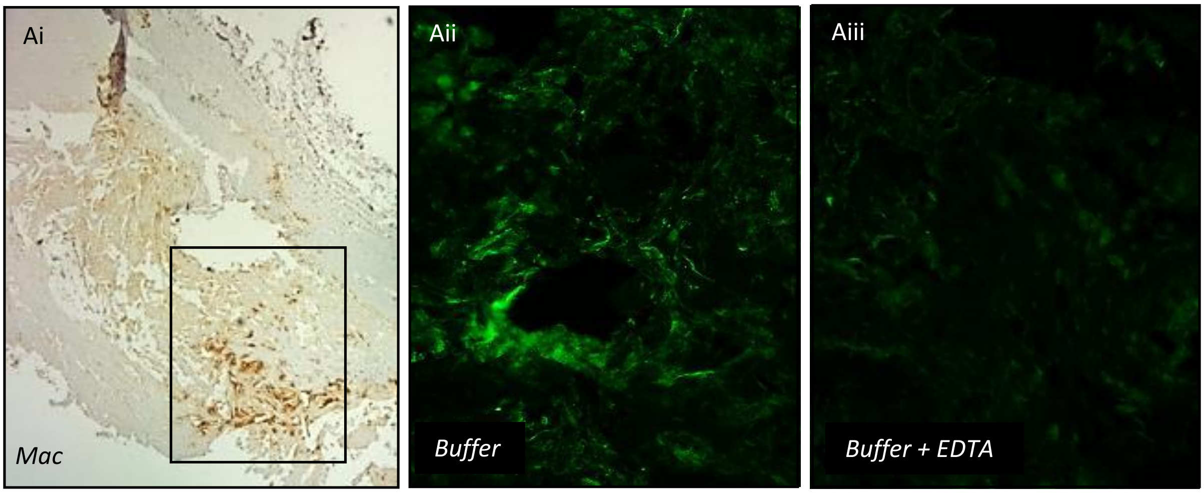

Supplemental Figure IV: Macrophage infiltration in human AAAs is associated with MMP activity
Human atherosclerotic abdominal aortic aneurysms demonstrating macrophage infiltration (Ai; macrophages labelled with CD68 – brown colour) which is associated with MMP activity as assessed by in situ zymography (green colour; Aii) as it can be retarded by a MMP inhibitor (EDTA; Aiii). Panels Aii, Aiii, are high power images from area indicated by black box in panel Ai.

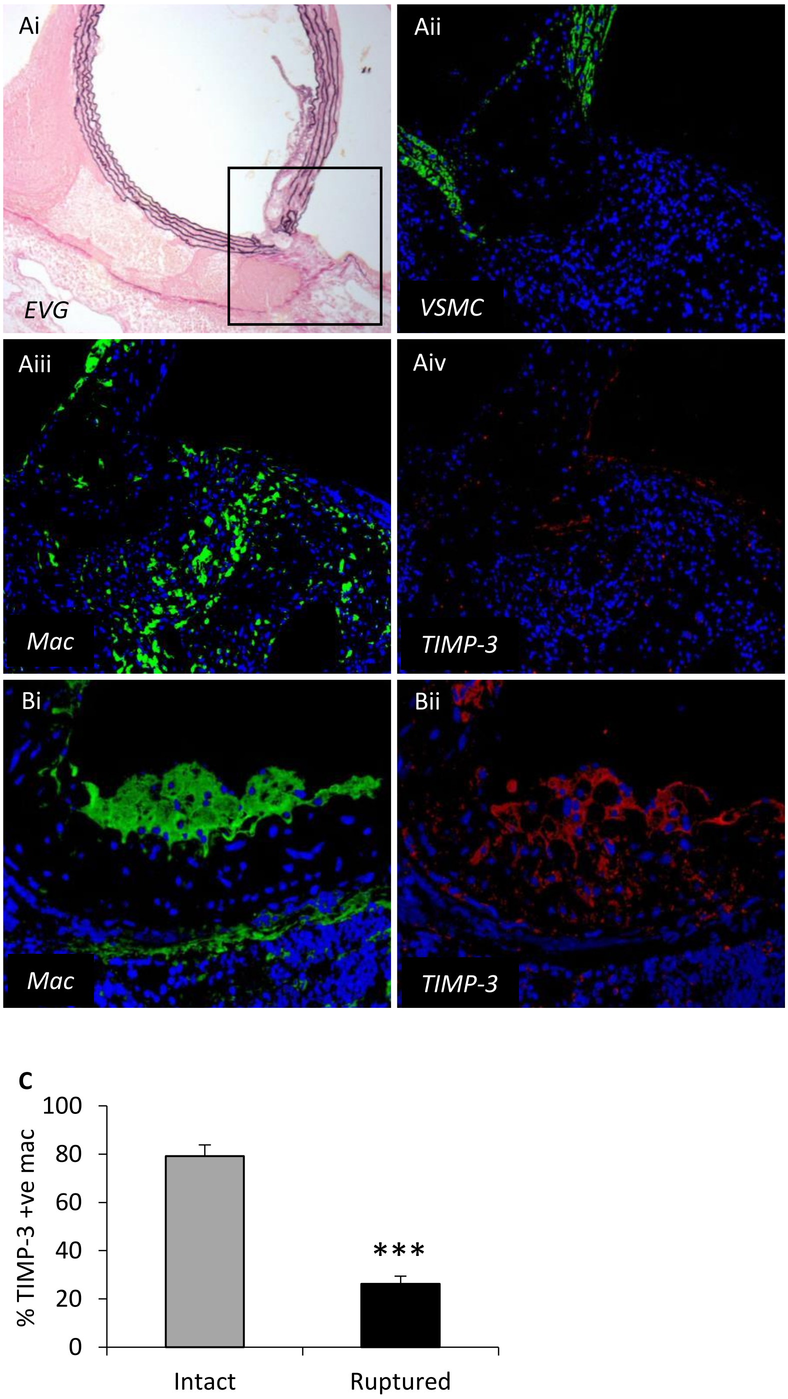

Supplemental Figure V: Diminished macrophage TIMP-3 expression associates with ruptured mouse aneurysms

(**A** and **B**) Analysis of atherosclerotic abdominal aortic aneurysms from Apoe$^{-/-}$ mice which have received Ang II and fed a high-fat diet for 4 weeks, and assessed by EVG for elastin breaks and atherosclerosis (Ai), α-smooth muscle actin for VSMC (green colour; Aii), F4/80 for macrophages (green colour; Aiii) and TIMP-3 (red colour; Aiv). Box in panel Ai represents high power field in panels Aii-Aiv. Non-aneurysmal arteries with abundant macrophages (green colour; Bi) and TIMP-3 (red colour; Bii). Nuclei are counterstained with DAPI (blue).

(**C**) Quantification of TIMP-3 positive macrophages (CD68) as assessed by immunofluorescence staining of ruptured and non-ruptured atherosclerotic AAAs from Ang II-infused 8 week high fat-fed Apoe$^{-/-}$ mice, n=6/group, ***P<0.000315, two-tailed Student's t-test, data expressed as mean±SEM.

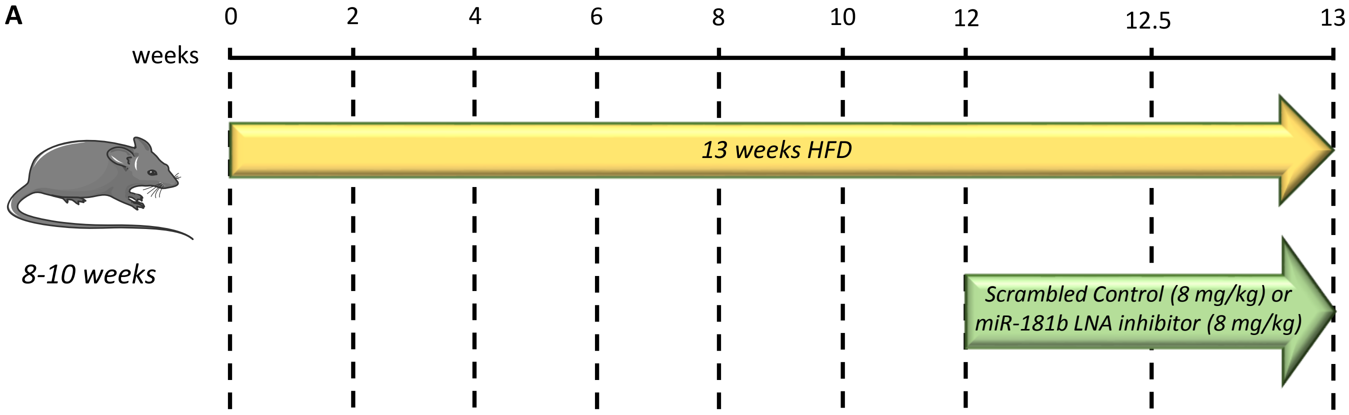

Supplemental Figure VI: Experimental design for atherosclerosis studies

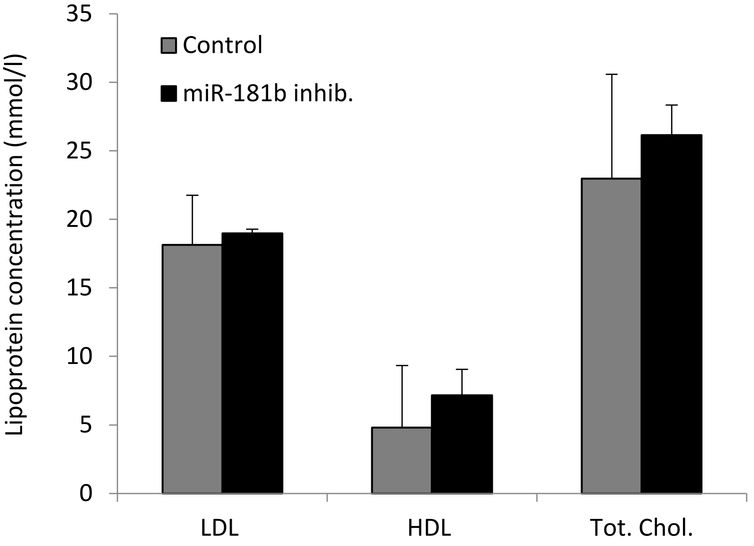

Supplemental Figure VII: Effect of miR-181b inhibition on plasma lipids in high-fat fed Apoe $^{-/-}$ mice
Quantification of plasma lipid profiles performed on terminal bloods drawn from the abdominal aorta of 13 week high-fat fed Apoe $^{-/-}$ mice after 1 week treatment of miR-181b inhibitor or a scrambled control, n=6/group, data expressed as mean±SEM.

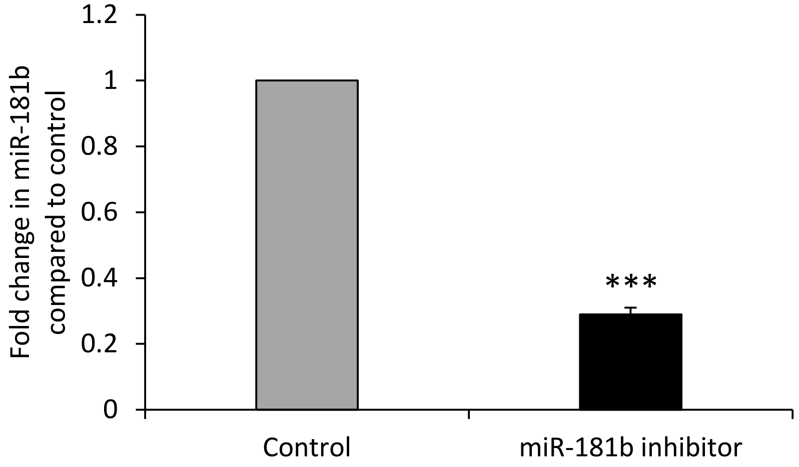

Supplemental Figure VIII: Effect of miR-181b inhibition on intra-plaque miR-181b expression
QPCR analysis and quantification of miR-181b expression in atherosclerotic brachiocephalic arteries from scr-control & LNA-miR-181b inhibitor treated mice, demonstrating that miR-181b expression was significantly reduced through miR-181b inhibition (n=6/group; ***p=0.000425 compared to scrambled control mice, two-tailed Student's *t*-test). Data is expressed as fold change in miR-181bexpression compared to controls, which is normalised to Scarna17 expression and set to 1.

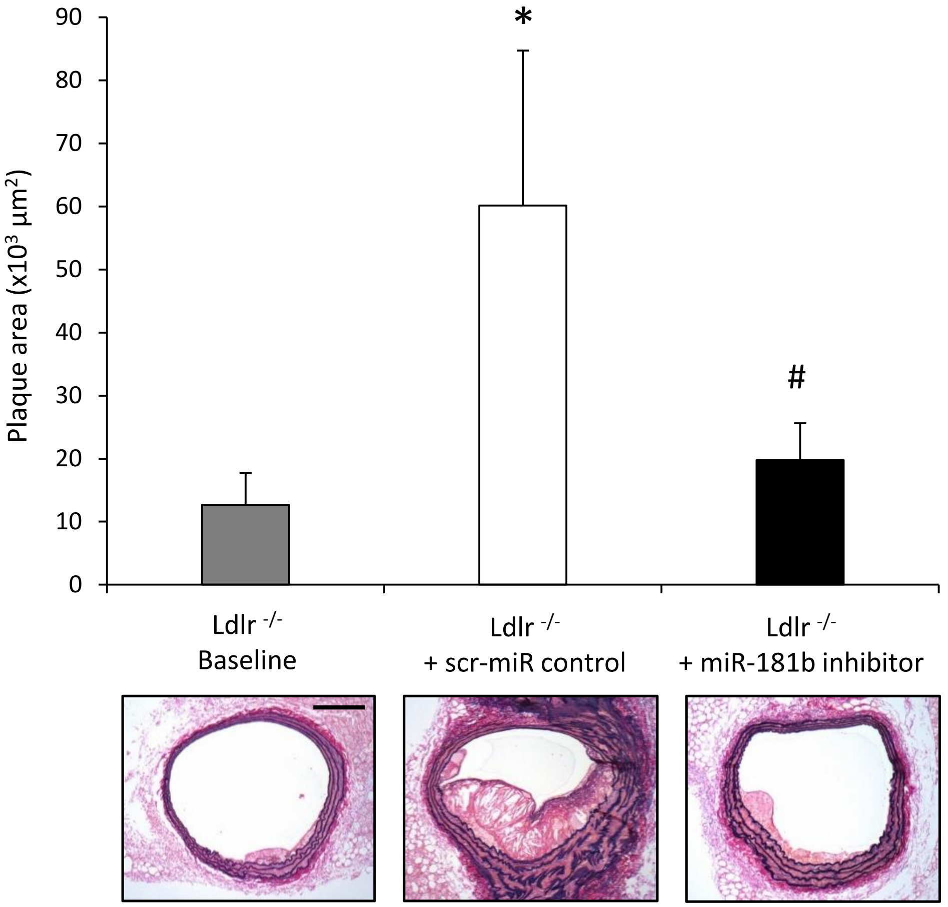

Supplemental Figure IX: miR-181b inhibition stabilises atherosclerotic plaques in hypercholesteroleamic Ldlr $^{-/-}$ mice

Representative images and quantification of plaque cross-sectional area in EVG-stained sections of plaques within the brachiocephalic artery, of Ldlr $^{-/-}$ mice high fat-fed for 8 weeks (baseline), or 12 weeks and scrambled (scr) control or miR-181b inhibitor-treated between 8 and 12 weeks of high fat-feeding, n=6-8/group, **P*<0.05 compared to Ldlr $^{-/-}$ baseline mice, $^{\#}$*P*<0.05 compared to Ldlr $^{-/-}$ scrambled control animals, ANOVA, scale bar represents 100µm and is applicable to all panels. Data represents the mean±SEM.

A. *Progression*

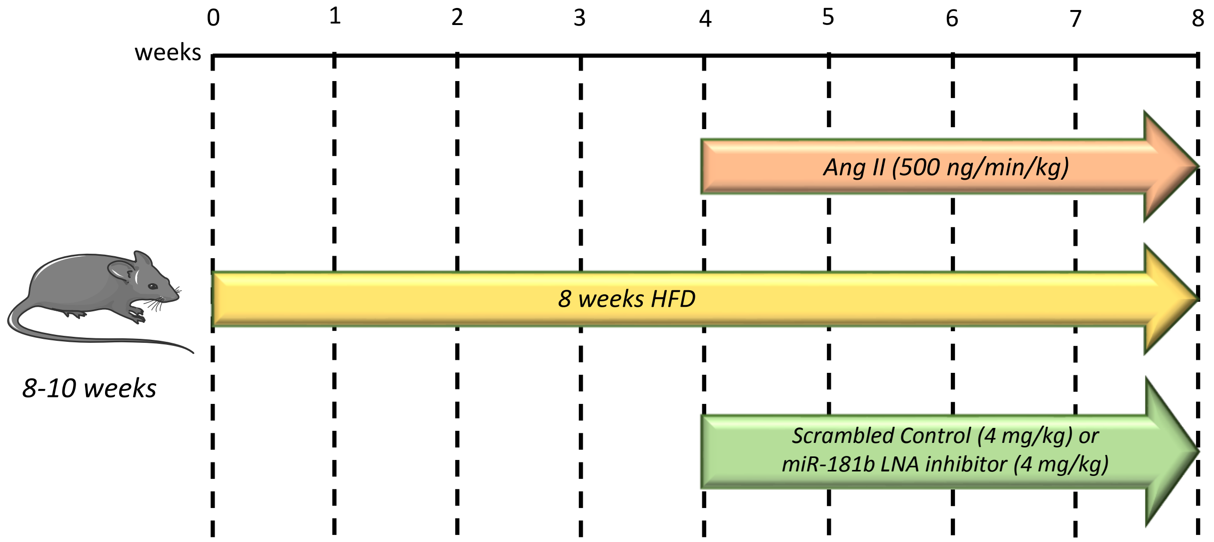

B. *Regression*

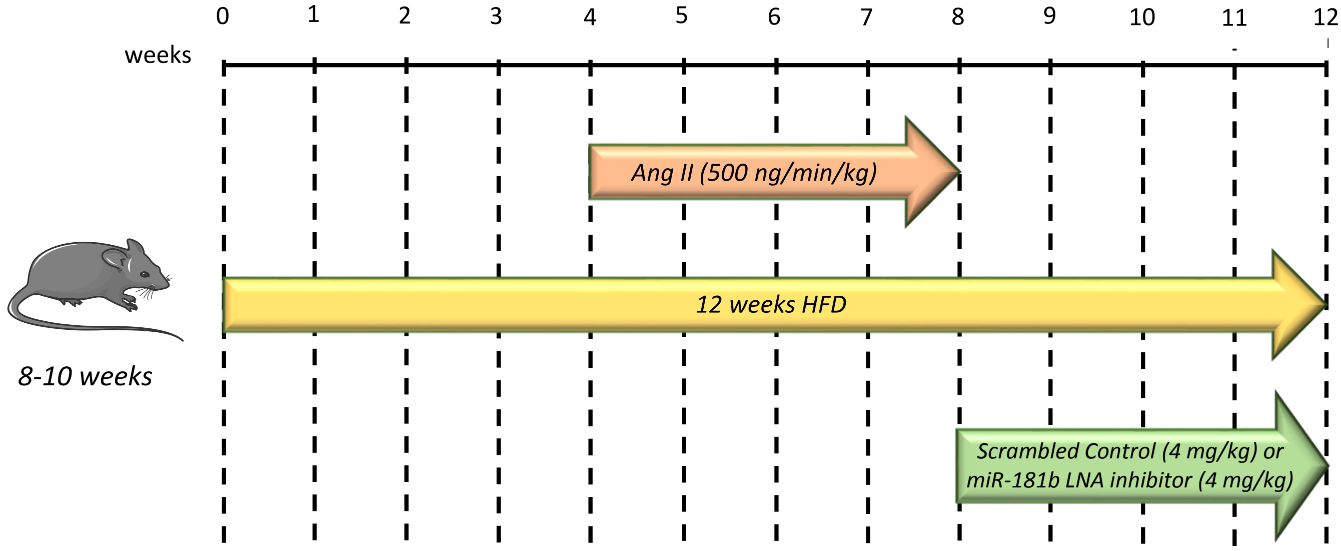

Supplemental Figure X: Experimental design for aneurysm studies

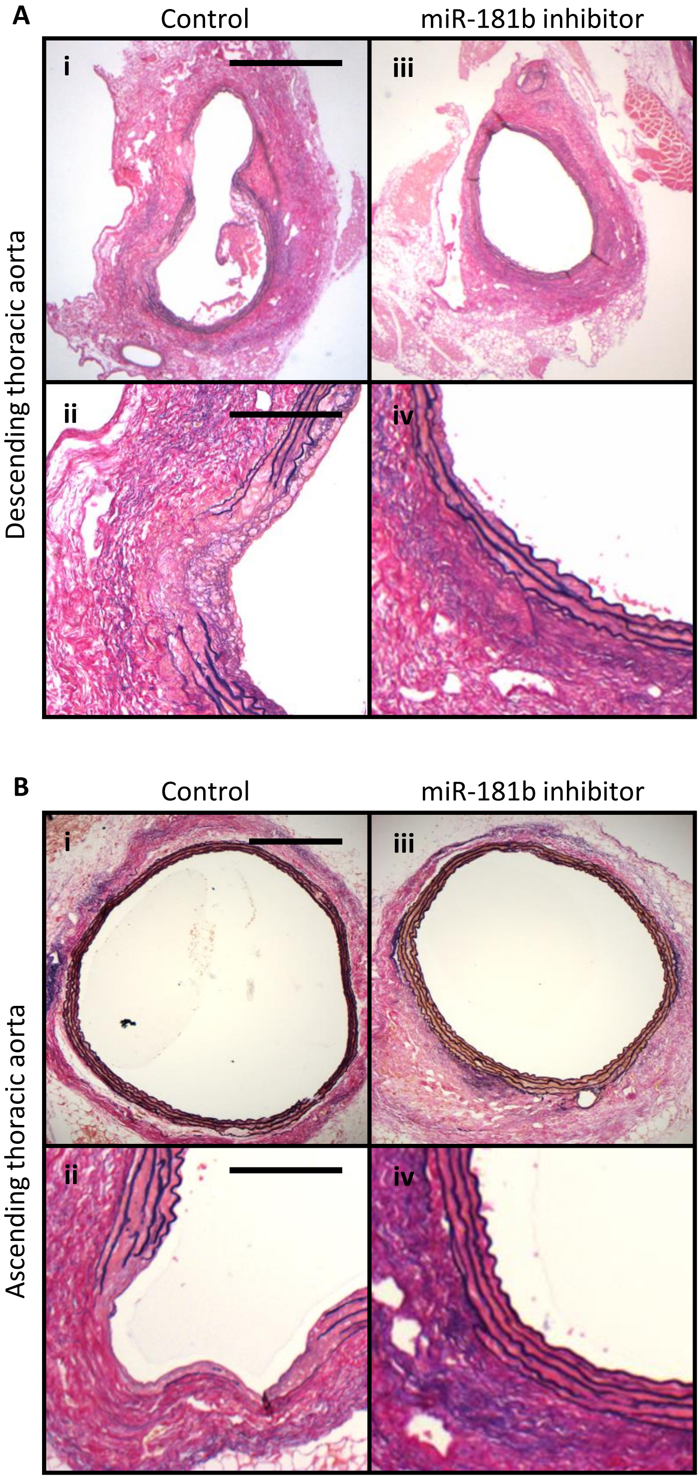

Supplemental Figure XI: miR-181b inhibition regulates elastin content and fragmentation at multiple aneurysmal sites in *Ldlr* $^{-/-}$ mice

Representative images of elastin van Gieson stained histological cross-sections of (**A**) descending and (**B**) ascending thoracic aortae from scrambled control and miR-181b inhibitor-treated *Ldlr* $^{-/-}$ mice, demonstrating differences in elastin (black) content and fragmentation, scale bar in Ai represents 200µm and is applicable to Ai and Aiii, scale bar in Aii represents 50µm and is applicable to Aii and Aiv, scale bar in Bi represents 100µm and is applicable to Bi and Biii, scale bar in Bii represents 50µm and is applicable to Bii and Biv.

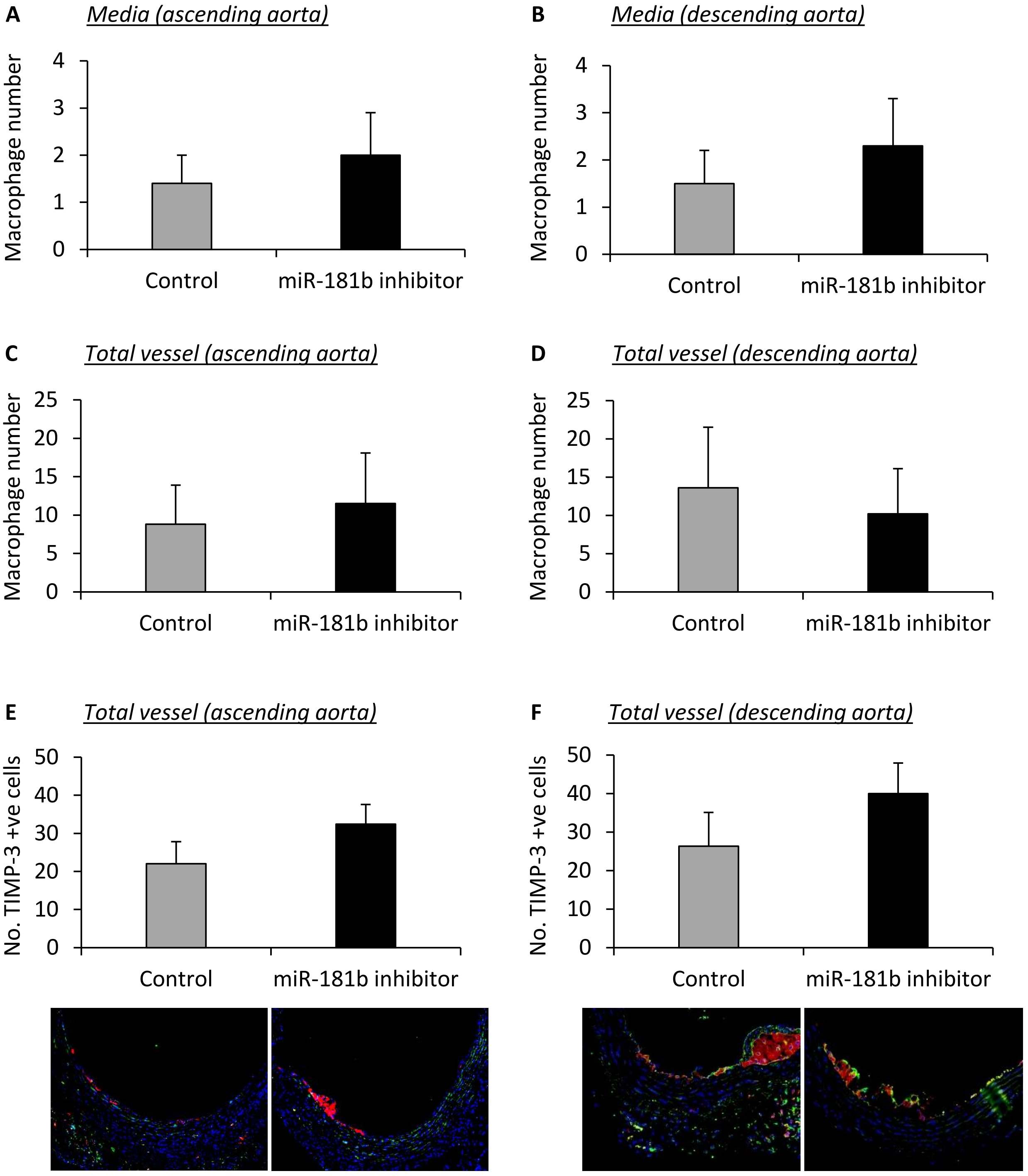

Supplemental Figure XII: Effect of miR-181b inhibition on macrophage content and TIMP-3 expression in ascending and descending aortas from *Apoe* $^{-/-}$ mice

Quantification and representative images of CD68 and TIMP-3 immuno-histochemical labelled cross-sections of (**A, C and E**) ascending and (**B, D and F**) descending thoracic aortae from scrambled control and miR-181b inhibitor-treated *Apoe* $^{-/-}$ mice. In representative images, red depicts CD68 (macrophages), green depicts TIMP-3, and blue indicates nuclei (Dapi).

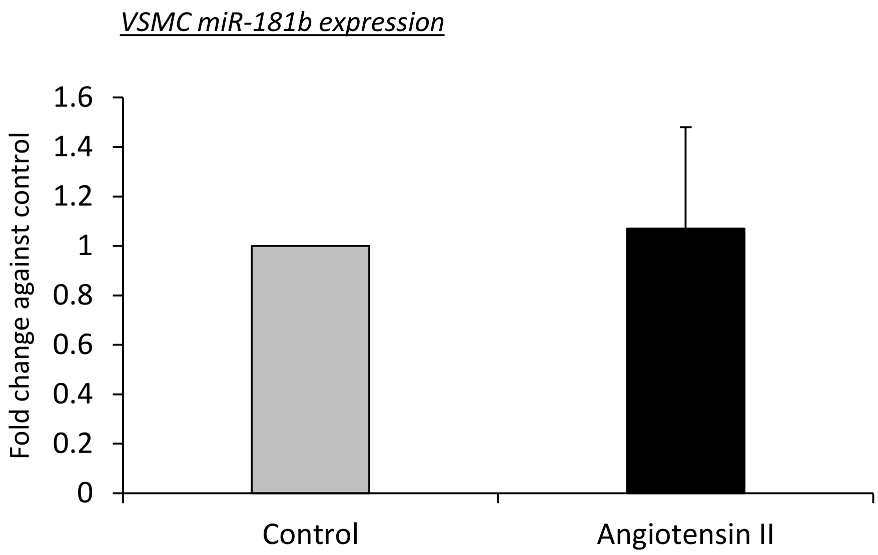

Supplemental Figure XIII: Angiotensin II does not regulate VSMC miR-181b expression
QPCR for miR-181b expression in VSMCs after 6 hour stimulation with angiotensin II (5µM) or PBS to serve as a control (n=4).

A *Intraobserver agreement*

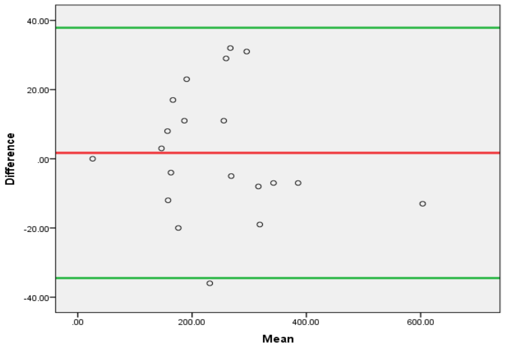

Coefficients[a]

Model		Unstandardized Coefficients		Standardized Coefficients	t	Sig.
		B	Std. Error	Beta		
1	(Constant)	7.720	9.804		0.787	0.441
	Mean	-0.025	0.036	-0.158	-0.679	0.506

a. Dependent Variable: Difference

B *Interobserver agreement*

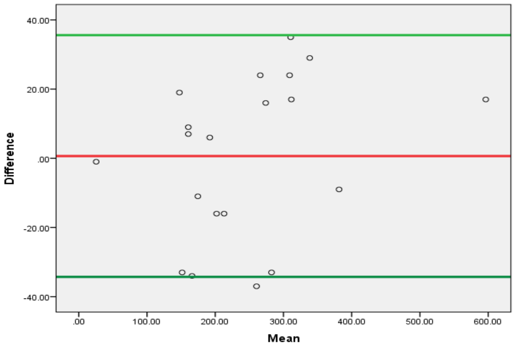

Coefficients[a]

Model		Unstandardized Coefficients		Standardized Coefficients	t	Sig.
		B	Std. Error	Beta		
1	(Constant)	-14.259	11.831		-1.205	0.244
	Mean	0.061	0.044	0.312	1.391	0.181

a. Dependent Variable: Difference

Supplemental Figure XIV: Assessment of intra- and inter-observer variability in histological assessment of intra-plaque TIMP-3 positive macrophages

Bland-Altman plots and associated statistical evaluation demonstrating good intra-observer (A) and inter-observer (B) agreement of TIMP-3 positive macrophages in stable and unstable human coronary artery atherosclerotic plaques. The red line indicate the mean, whilst the green lines depict upper and lower confidence levels.

Supplemental Table I: List of primary antibodies

Antibody	Application	Dilution	Company	Code
Alpha-Smooth Muscle Actin	IHC	1/400	Sigma	A2547
β-Actin	WB	1/10000	Sigma	A5316
Cleaved Caspase 3	IHC	1/100	Cell Signalling	#9664
CD68	IHC (Ms)	1/100	Santa Cruz	sc-7084
CD68	IHC (Hm)	1/100	Dako	M0814
Elastin	WB	1/200	Santa Cruz	sc-17580
GSLII	IHC	1/200	Vector Labs	B-1215
GM-CSF	IHC	1/50	R&D Systems	MAB215
PCNA	IHC	1/100	Cell Signalling	#13110
TIMP-3	IHC	1/200	Millipore	MAB3318
TIMP-3	IHC / WB	1/200 / 1/1000	Abcam	ab39184

Supplemental Table II: List of Q-PCR primers

Gene/Primer ID	Species		Sequence
Acta2	Ms	Forward	TTCGTGACTACTGCCGAGCGTG
		Reverse	GAGAGTCTCTGGGCAGCGGAAG
ActB	Ms	Forward	AGGCGGACTGTTACTGAGCTGC
		Reverse	CTGTCGCCTTCACCGTTCCAGT
Bax	Ms	Forward	CTGGATCCAAGACCAGGGTG
		Reverse	CCTTTCCCCTTCCCCCATTC
Cd68	Ms	Forward	TGGCGGTGGAATACAATGTGTC
		Reverse	GACTGGTCACGGTTGCAAGAGA
p53	Ms	Forward	AGGATGCCCATGCTACAGAG
		Reverse	TGAGTGGATCCTGGGGATTGTG
TIMP3	Hm	Forward	CTTCCGAGAGTCTCTGTGGCCTTA
		Reverse	CTCGTTCTTGGAAGTCACAAAGCA
Timp3	Ms	Forward	CACATCAAGGTGCCATTCAGGTAG
		Reverse	GTTCTCTCCTCCTCAACCCAAACA
